# Analysis of codon usage patterns in 48 *Aconitum* species

**DOI:** 10.1186/s12864-023-09650-5

**Published:** 2023-11-22

**Authors:** Meihua Yang, Jiahao Liu, Wanqing Yang, Zhen Li, Yonglin Hai, Baozhong Duan, Haizhu Zhang, Xiaoli Yang, Conglong Xia

**Affiliations:** 1 College of Pharmaceutical Science, Dali University, Dali, Yunnan 671000 China; 2 Key Laboratory of Yunnan Provincial Higher Education Institutions for Development of Yunnan Daodi Medicinal Materials Resources, Dali, Yunnan 671000 China; 3 Western Yunnan Traditional Chinese Medicine and Ethnic Drug Engineering Center, Dali, Yunnan 671000 China

**Keywords:** *Aconitum*, Optimal codon, Codon usage bias, Complete genome

## Abstract

**Background:**

The *Aconitum* genus is a crucial member of the Ranunculaceae family. There are 350 *Aconitum* species worldwide, with about 170 species found in China. These species are known for their various pharmacological effects and are commonly used to treat joint pain, cold abdominal pain, and other ailments. Codon usage bias (CUB) analysis contributes to evolutionary relationships and phylogeny. Based on protein-coding sequences (PCGs), we selected 48 species of *Aconitum* for CUB analysis.

**Results:**

The results revealed that *Aconitum* species had less than 50% GC content. Furthermore, the distribution of GC content was irregular and followed a trend of GC_1_ > GC_2_ > GC_3_, indicating a bias towards A/T bases. The relative synonymous codon usage (RSCU) heat map revealed the presence of conservative codons with slight variations within the genus. The effective number of codons (ENC)-Plot and the parity rule 2 (PR2)-bias plot analysis indicate that natural selection is the primary factor influencing the variation in codon usage. As a result, we screened various optimal codons and found that A/T bases were preferred as the last codon. Furthermore, our Maximum Likelihood (ML) analysis based on PCGs among 48 *Aconitum* species yielded results consistent with those obtained from complete chloroplast (cp.) genome data. This suggests that analyzing mutation in PCGs is an efficient method for demonstrating the phylogeny of species at the genus level.

**Conclusions:**

The CUB analysis of 48 species of *Aconitum* was mainly influenced by natural selection. This study reveals the CUB pattern of *Aconitum* and lays the foundation for future genetic modification and phylogenetic analyses.

**Supplementary Information:**

The online version contains supplementary material available at 10.1186/s12864-023-09650-5.

## Introduction

The Ranunculaceae family includes the *Aconitum* genus, which consists of around 350 species primarily found in Asia, with Europe and North America following closely behind [1]. China alone has about 200 species of *Aconitum* [2], with the majority located in the Hengduan Mountain Region of Northern Yunnan, Western Sichuan, and Eastern Tibet. This region is home to approximately 76 medicinal species of *Aconitum* [3]. The 2020 Edition of the Chinese Pharmacopoeia [4] lists *A. carmichaelii* and *A. kusnezoffii* as the fundamental medicinal ingredients for ‘Chuanwu’ and ‘Caowu.‘ In addition, it was found that TCM is used to treat various syndromes, such as straight anesthesia, joint pain, wind, cold and wet paralysis, cold and abdominal pain, and pain caused by cold herniation.

At the same time, Mongolian medicine is primarily utilized in clinical settings to treat acute stinging pain, plague, and fattening cold, among other ailments [5]. However, the applications of these remedies vary. For example, *A. episcopale* is known for its anti-alcoholic properties and ability to detoxify opium [6], while *A. sinomontanum* contains aconitine and is commonly used for therapeutic analgesia [7]. Furthermore, *A. brachypodum* and *A. nagarum* Stapf var. *heterotrichum* are widely utilized species for their exceptional medicinal properties. However, the excessive collection of these species by digging up their tubers has led to a decline in their population and caused significant harm to their natural habitat. As a result, they have been classified as endangered species in the China plant red data book [8].

Chloroplasts play a crucial role in photosynthesis by providing energy to the plant and facilitating the synthesis of secondary metabolites. In addition, it is an essential unit of cytoplasmic inheritance in plants [9]. Chloroplast genomes are characterized by their small number, highly conserved, and low rate of evolution. In addition, their sequences are readily available [10]. Codons are essential in forming a junction between the nucleic acids and the proteins to transmit genetic information [11]. It is worth noting that only two amino acids, methionine (Met) [12] and tryptophan (Trp) [13], are encoded by a single codon, while 2–6 codons encode the rest. This phenomenon of different codons encoding the same amino acid is known as synonymous codons [14]. However, when looking at how amino acids are encoded in other genes or genomes, there is variation in the frequent usage of these synonymous codons, known as codon usage bias (CUB) [15]. Several studies have demonstrated the significance of codon preference in the expression of foreign genes [16]. Therefore, investigating the codon usage patterns of organisms and the factors that impact the molecular mechanism of plant gene expression can enhance the efficiency of genetic transformation.

With the continuous progress of science and technology, more and more studies have been conducted on the codons of plant chloroplast genomes. As an important medicinal plant, the genus *Aconitum* has a wide range of species, and the codon analysis of previous studies was limited to a single species. Therefore, in this study, we analyzed the codon usage patterns of 48 *Aconitum* species and compared the GC content, ENC-Plot, PR2-Plot, optimal codons, and the RSCU heatmap. In addition, a phylogenetic tree (ML) was constructed, providing a feasible method for analyzing specie’s evolution.

## Results

### Analysis of codon base composition

**Fig. 1:**
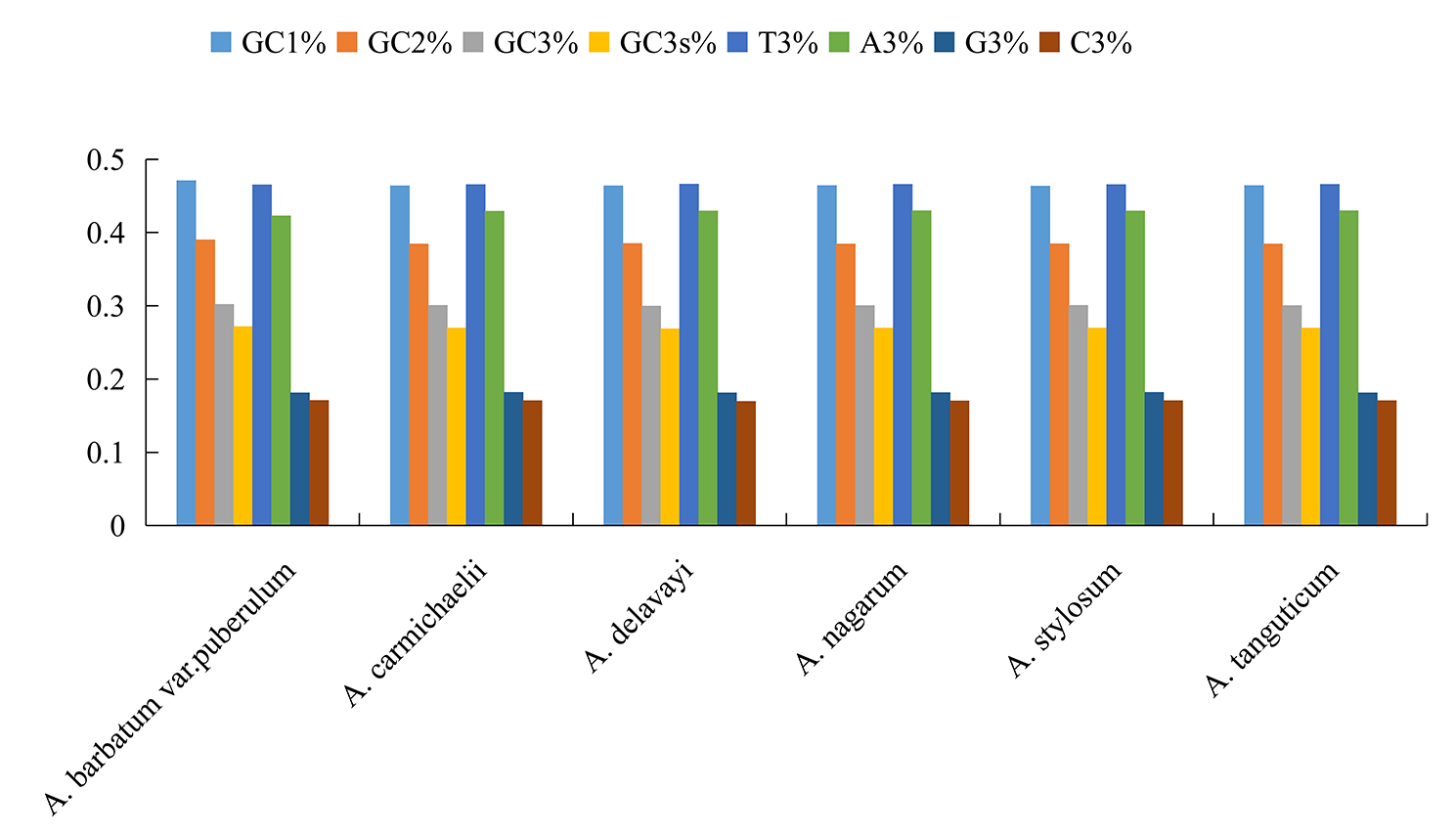
Comparative analysis of GC contents and nucleotide distribution for six *Aconitum* species

The GC content at the three codon positions (GC_1_/GC_2_/GC_3_), as well as the GC nucleotide content in the synonymous codon for the same amino acid at codon 3, expressed as GC_3S_, were calculated using the screened CDS (Supplementary Table 2). Higher AT content and lower GC content were observed in 48 species of *Aconitum*. At the same time, the GC content of all three loci was below 50%, suggesting a preference for A/T bases and A/T ending codons in *Aconitum* (Fig. 1). Among the studied species, *A. barbatum* var. *puberulum* had the highest GC content of 47.12%, while *A. barbatum* had the lowest GC content of 44.34%. In addition, *A. barbatum* var. *puberulum* had the highest GC_1_ content while *A. barbatum* had the lowest. On the other hand, *A. barbatum* had the highest GC_2_ content while *A. barbatum* var. *puberulum* had the lowest GC_3_ and GC_3s_ values among the studied species, which suggests that the codons of this species are not conserved and have been more active during the evolutionary process (Supplementary Table 2) [17].

In addition, the GC contents of the three loci were not balanced, and the distribution trend of *Aconitum* was GC_1_ > GC_2_ > GC_3_. The GC contents of *Aconitum* were similar, and the A/T base contents showed higher contents than G/C. It was also observed that there were more A/T bases at the end of dicotyledonous plants and more G/C bases at the end of monocotyledonous plants. This observation confirms the codon identity of the sequence composition of *Aconitum*.

### Codon usage patterns across the *Aconitum*

**Fig. 2:**
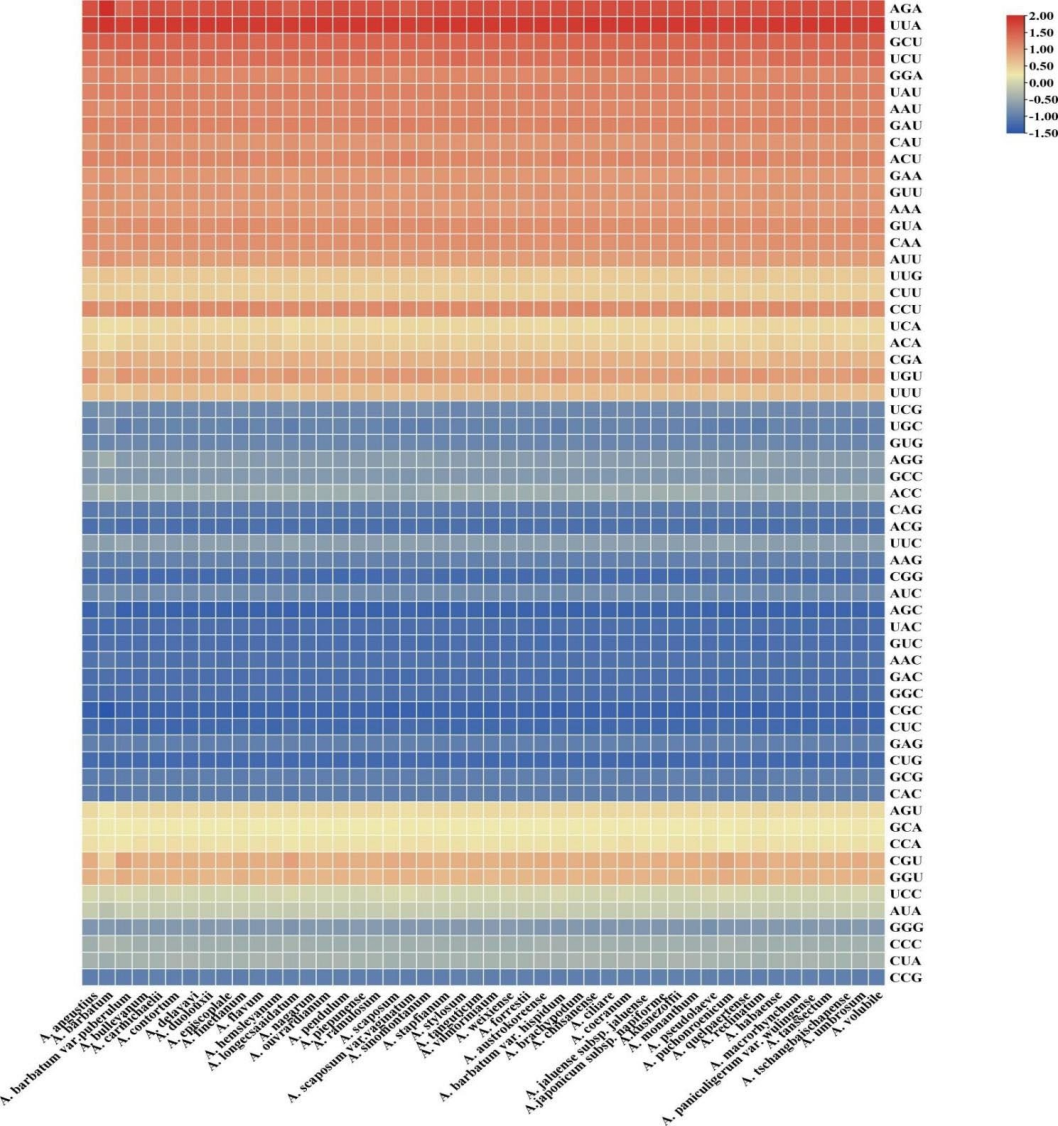
Hierarchical clustering of heat map for 48 *Aconitum* using RSCU values. The color and the degree of intensity indicate the RSCU value, and it varies from blue to red with a low value to a high value of RSCU

The cp. genomes of the 48 *Aconitum* species had a total of 36 codons (RSCU > 1), of which 35 ended in A/U (97.22%), suggesting that *Aconitum* has a preference for these codons. Meanwhile, the RSCU values of CDS were similar in *Aconitum* (Supplemental Table 3). However, there were subtle differences in RSCU values between these species, suggesting relative conservation between species during the evolution of *Aconitum* (Fig. 2). In addition, we observed higher RSCU values at the third codon position ending in the A/U base, suggesting more significant codon usage bias.

### Identification of optimal codon

**Fig. 3:**
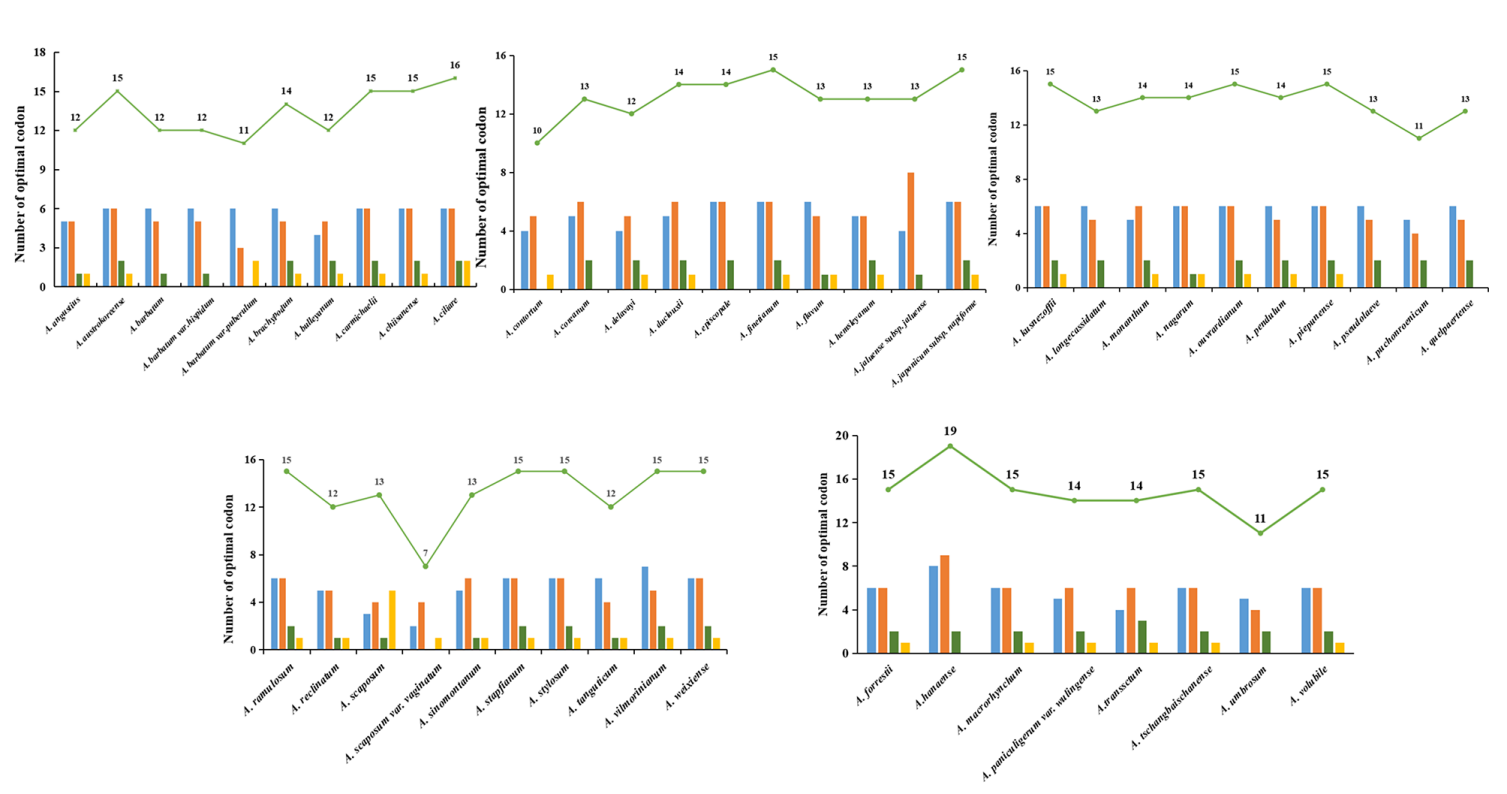
Analysis of optimal codon number in 48 *Aconitum* plants

Each CDS was sorted according to the ENC value, and the genes with 10% at both ends were selected to create high and low-expression libraries, respectively. The RSCU value and ∆RSCU in each library were calculated, and based on the ∆RSCU value, the optimal codon for the genus *Aconitum* to be evaluated was determined. *A. ciliare* chose 16 optimal codons, while *A. scaposum* var. *vaginatum* had seven optimal codons. Meanwhile, 15 optimal codons were identified in 19 more species (Fig. 3). Many exogenous systems have been introduced with optimal codon usage [18–20]. We found optimal codons ending in A/T in 48 species containing three common optimal codons, including UGA, GGA, and UAU (Supplemental Table 4). This phenomenon has important implications for the expression of exogenous proteins.

### Analysis of sources of codon usage bias

#### Correlation analysis

**Fig. 4:**
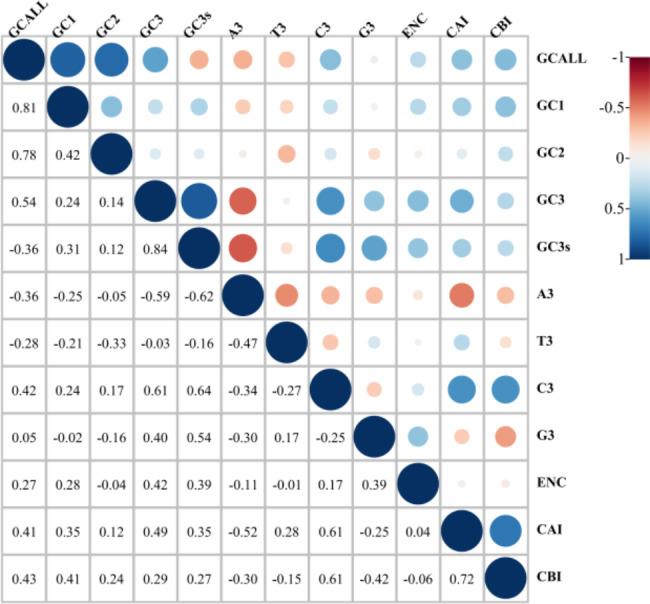
Correlation analysis among the parameters of *A. forrestii* codon usage pattern

Correlation analysis assessed the relationship between codon-related indicators such as third codon position, ENC value, and GC content. There were differences in the base composition of the three codon positions, in which the GC_ALL_ content was significantly positively correlated with GC_1_, GC_2_, and GC_3_. However, the content of GC_3_ showed no significant correlation with GC_1_ or GC_2_, suggesting differences between GC_1_/GC_2_ and GC_3_. (Fig. 4, Supplementary Table 2). Except for *A. carmichaelii* and *A. hemsleyanum*, the results also showed that ENC values were significantly correlated with GC_3_, suggesting that GC_3_ may be necessary for contributing to codon usage bias in the cp. genome (Supplementary Table 5).

#### ENC-Plot analysis

**Fig. 5:**
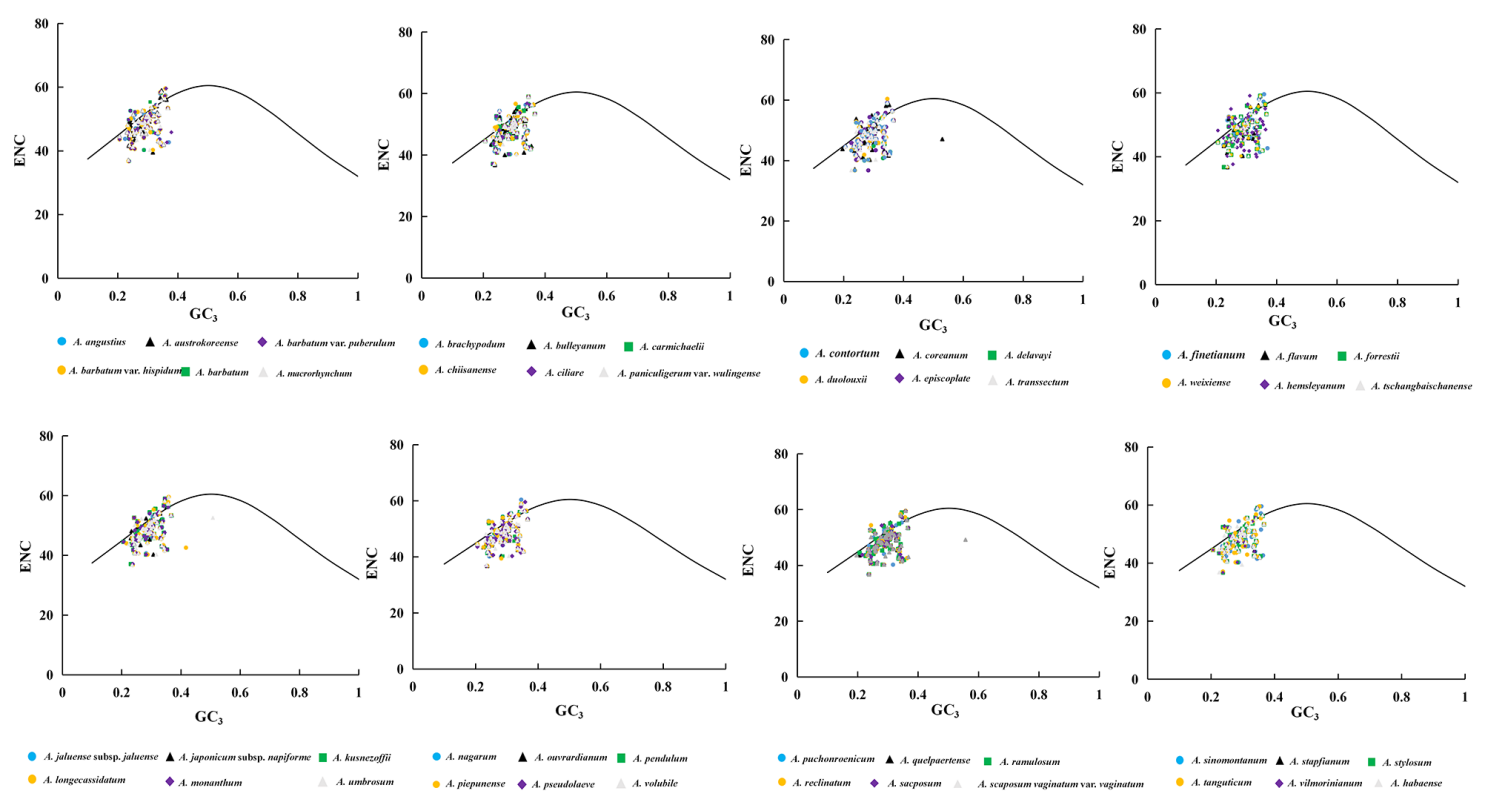
ENC-GC_3_ plots of 48 *Aconitum* species

By exploring the relationship between ENC values and GC_3_, we can analyze the factors that influence the gene or genomic codon usage pattern. Figure 5 shows a similar distribution of ENC values and GC_3_ values for the 48 *Aconitum* species. The analysis shows that the ENC values of most genes are below ENCexp, and only a few points are close to the curve. In addition, most of the genes are located below the arc, which suggests that natural selection plays an essential role in codon usage patterns.

In addition, the frequency distribution table of (ENCexp-ENCobs)/ENCexp in genes was utilized in this study to investigate the differences between the observed ENCs and the expected values (Table 1). The peak distribution of genes ranged from − 0.05 to 0.15, with more than 50% falling within this range. These values indicate gene mutations also affect codon usage bias in the cp. genome. The results of the analysis are consistent with Fig. 5.

**Table 1:** Frequency distribution of ENC ratios

Species	-0.15~-0.05	-0.05 ~ 0.05	0.05 ~ 0.15	0.15 ~ 0.25	0.25 ~ 0.35
*A. angustius*	0.038	0.396	0.485	0.151	0
*A. austrokoreense*	0.037	0.444	0.407	0.926	0.019
*A. barbatum*	0.057	0.340	0.453	0.151	0
*A. barbatum* var. *hispidum*	0.037	0.389	0.426	0.148	0
*A. barbatum* var.*puberulum*	0.075	0.377	0.396	0.151	0
*A. brachypodum*	0.075	0.340	0.453	0.132	0
*A. bulleyanum*	0.075	0.377	0.377	0.151	0
*A. carmichaelii*	0.057	0.396	0.485	0.132	0
*A. chiisanense*	0.057	0.485	0.396	0.132	0
*A. ciliare*	0.076	0.377	0.396	0.151	0
*A. coeranum*	0.057	0.321	0.434	0.170	0.019
*A. contortum*	0.057	0.396	0.485	0.113	0.019
*A. delavayi*	0.057	0.396	0.396	0.132	0.019
*A. duolouxii*	0.057	0.340	0.453	0.151	0
*A. episcoplale*	0.057	0.358	0.434	0.132	0.019
*A. finetianum*	0.075	0.340	0.434	0.151	0
*A. flavum*	0.057	0.358	0.453	0.132	0
*A. forrestii*	0.057	0.377	0.485	0.151	0
*A. hemsleyanum*	0.094	0.321	0.485	0.132	0.038
*A. jaluense* subsp. *jaluense*	0.057	0.377	0.434	0.132	0
*A. japonicum* subsp. *napiforme*	0.038	0.485	0.485	0.132	0
*A. kusnezoffii*	0.057	0.378	0.434	0.132	0
*A. longecsaaidatum*	0.038	0.385	0.404	0.154	0.019
*A. monanthum*	0.038	0.378	0.453	0.132	0
*A. nagarum*	0.075	0.321	0.453	0.151	0
*A. ouvrardianum*	0.075	0.302	0.472	0.151	0
*A. pendulum*	0.075	0.321	0.472	0.132	0
*A. piepunense*	0.075	0.340	0.434	0.151	0
*A. pseudolaeve*	0.019	0.396	0.434	0.151	0
*A. puchonroenicum*	0.038	0.377	0.485	0.170	0
*A. quelpaertense*	0.057	0.340	0.434	0.170	0
*A. ramulosum*	0.075	0.321	0.453	0.151	0
*A. reclinatum*	0.077	0.327	0.462	0.135	0
*A. scaposum*	0.057	0.358	0.434	0.151	0
*A. scaposum* var.*vaginatum*	0.058	0.327	0.423	0.192	0
*A. sinomontanum*	0.094	0.245	0.528	0.132	0
*A. stapfianum*	0.057	0.358	0.434	0.151	0
*A. stylosum*	0.057	0.396	0.396	0.151	0
*A. tanguticum*	0.057	0.396	0.485	0.132	0
*A. vilmoriniaum*	0.057	0.340	0.453	0.151	0
*A. weixiense*	0.057	0.358	0.434	0.151	0
A. *habaense*	0.039	0.434	0.377	0.151	0
A. *macrorhynchum*	0.038	0.434	0.396	0.132	0
A. *paniculigerum var. wulingense*	0.057	0.485	0.377	0.151	0
*A. transsectum*	0.038	0.396	0.485	0.151	0
*A. tschangbaischanense*	0.057	0.396	0.485	0.132	0
*A. umbrosum*	0.038	0.358	0.453	0.151	0
*A. volubile*	0.038	0.434	0.396	0.132	0

PR2-bias plot analysis

**Fig. 6:**
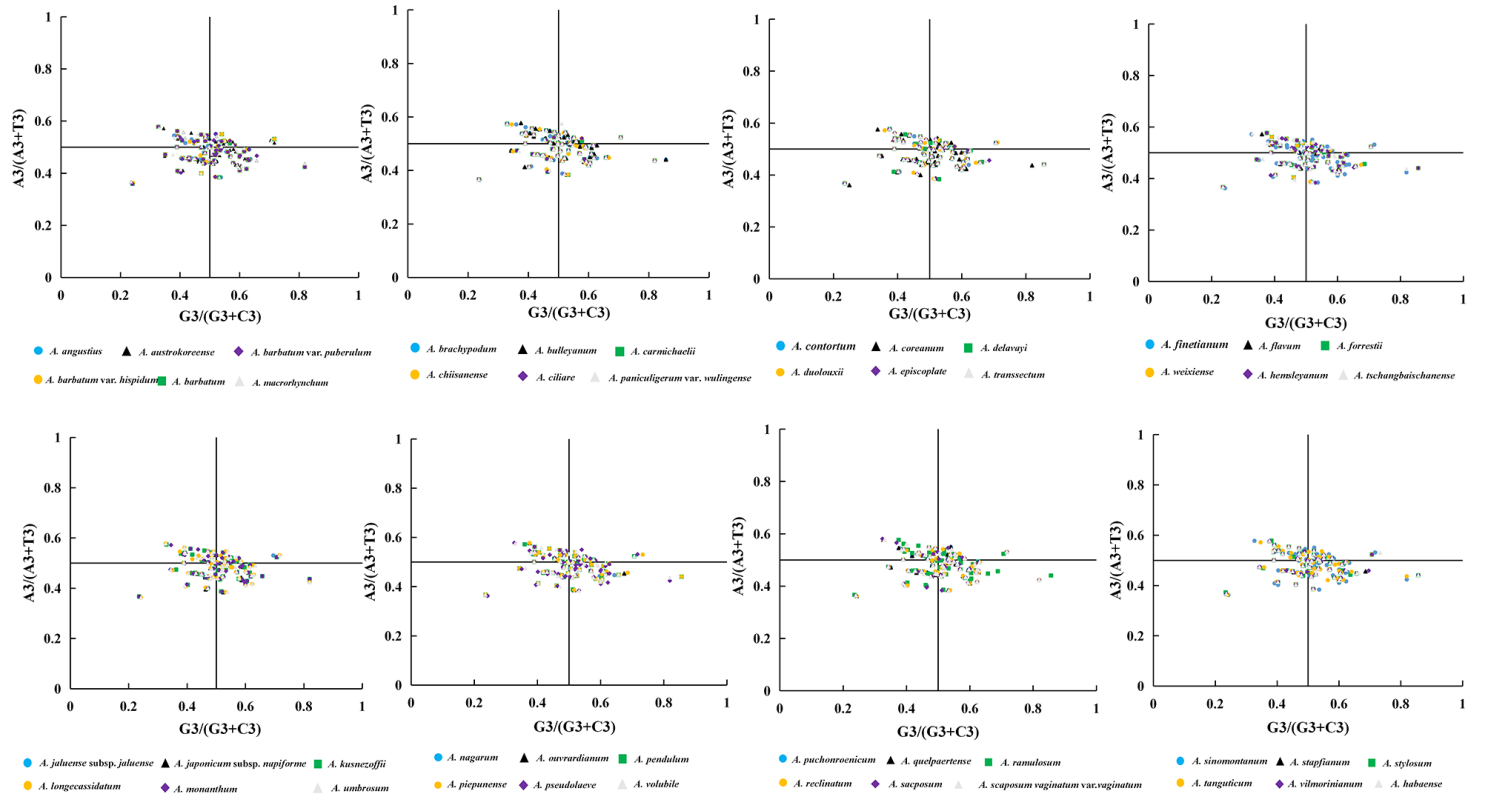
PR2-Plot of 48 *Aconitum* species

To further validate the factors affecting the bias in using PCGs for *Aconitum*, the correlation between purines (A/G) and pyrimidines (C/T) at codon position three was analyzed using PR2 bias plots. If the codon is biased in favor of G/C, the codon bias occurs mainly due to mutation. On the other hand, if natural selection dominates, it does not necessarily lead to proportional use of G/C. In the present study, it was observed that most of the genes were distributed in the lower part of the plane (Fig. 6), which implies that unbalanced use of the third codon is prevalent in the genes of *Aconitum* and that there is an A/T bias in the chloroplast genome. The base imbalance suggests a preference for natural selection as the dominant factor, with the influence of mutational pressure and other factors.

#### Genomic variation analysis

The cp. genome of *A. forrestii* was used as a reference sequence and compared with the cp. genomes of other *Aconitum* species to determine the overall degree of variation in these genomes. The results indicate that the non-coding regions of the cp. genome sequence exhibit more significant variation than the conserved protein-coding regions.

**Fig. 7:**
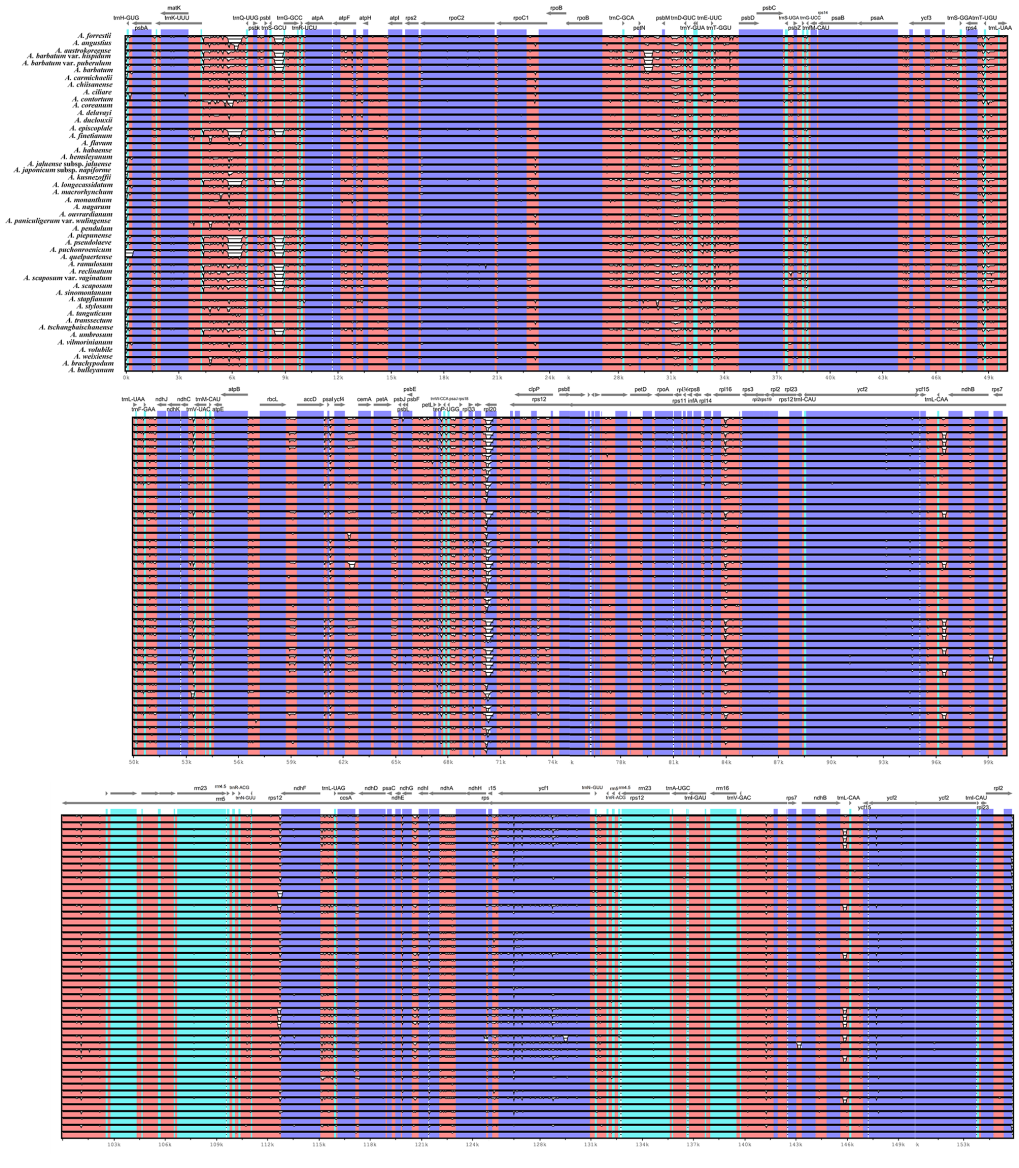
Visualization alignment of chloroplast genome in *Aconitum* plant

In addition, the rRNA gene regions were highly conserved, with only a few variants observed. Among the genes in the cp. genome, *ycf1, ycf2*, and *rpl20*, exhibited the most differential expression, while the rest were highly conserved. Moreover, the study found that variants in intergenic spacer regions (IGS) were more common than in intergenic other areas. Specifically, the *petN-psbM*, *matK-trnQ-UUQ*, *trns-GCU*, and *trnL-CAA-ndhB* intervals showed significant variation in the comparative analysis (Fig. 7). These regions showed significant variation during comparative analysis, and specific fragments could be developed from them, providing a new locus resource for molecular identification of *Aconitum* plants.

#### SNP

**Fig. 8:**
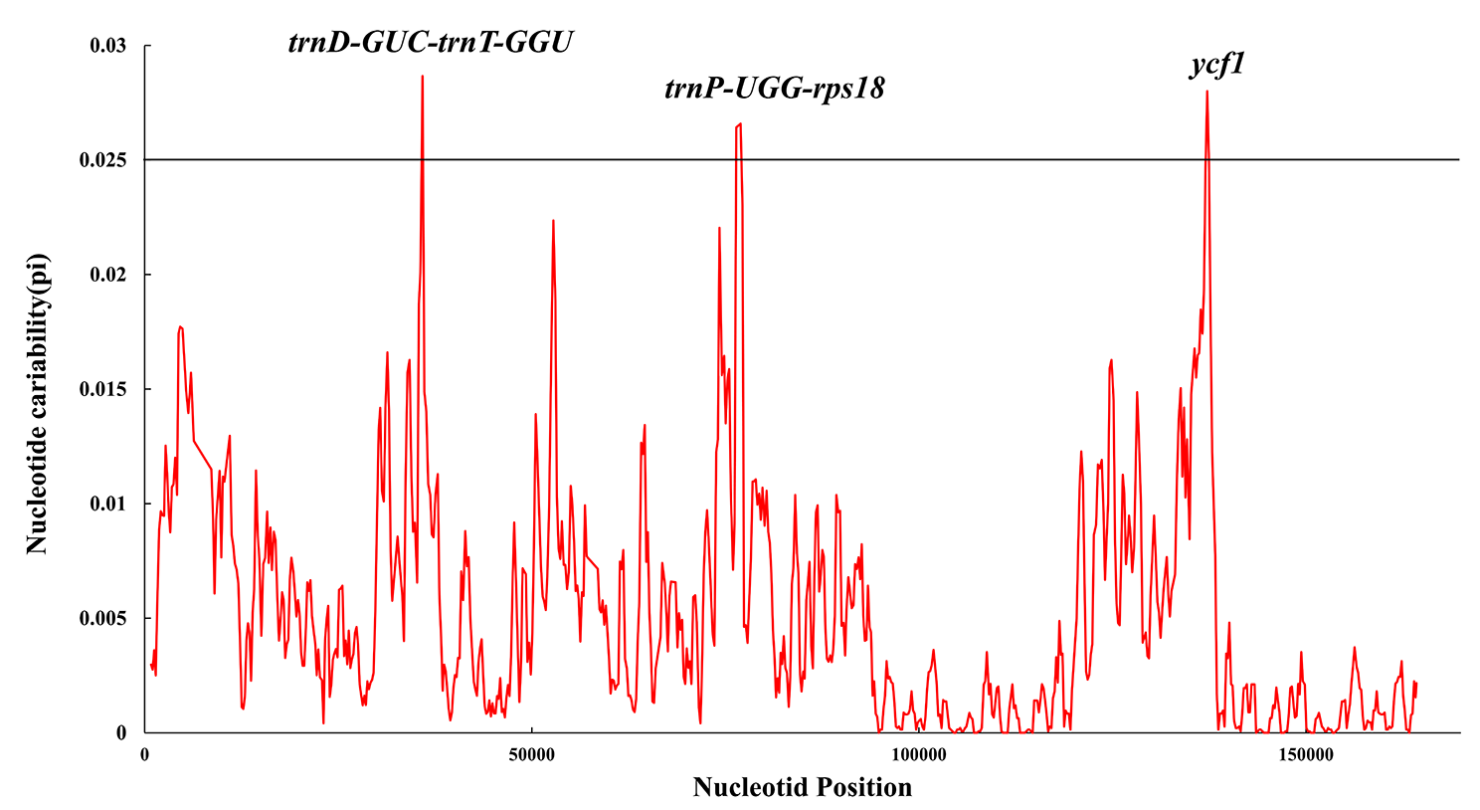
The nucleotide variability

This study aimed to assess the extent of diversity among 48 species of *Aconitum* through sliding window analysis. The findings revealed that no gene rearrangement was detected. However, upon further screening analysis, it was observed that two gene spacer regions (*trnD-GUC-trnT-GGU*, *trnP-UGG-rps18*) and one protein-coding gene region (*ycf1*) had higher Pi values (> 0.0025) (Fig. 8). The highly variable loci found in *Aconitum* plants can offer significant insights into species identification, phylogenetic relationships, and genetic diversity research. In addition, PCR primer information can be found in **Supplementary 6**.

#### Phylogenetic tree analysis

**Fig. 9:**
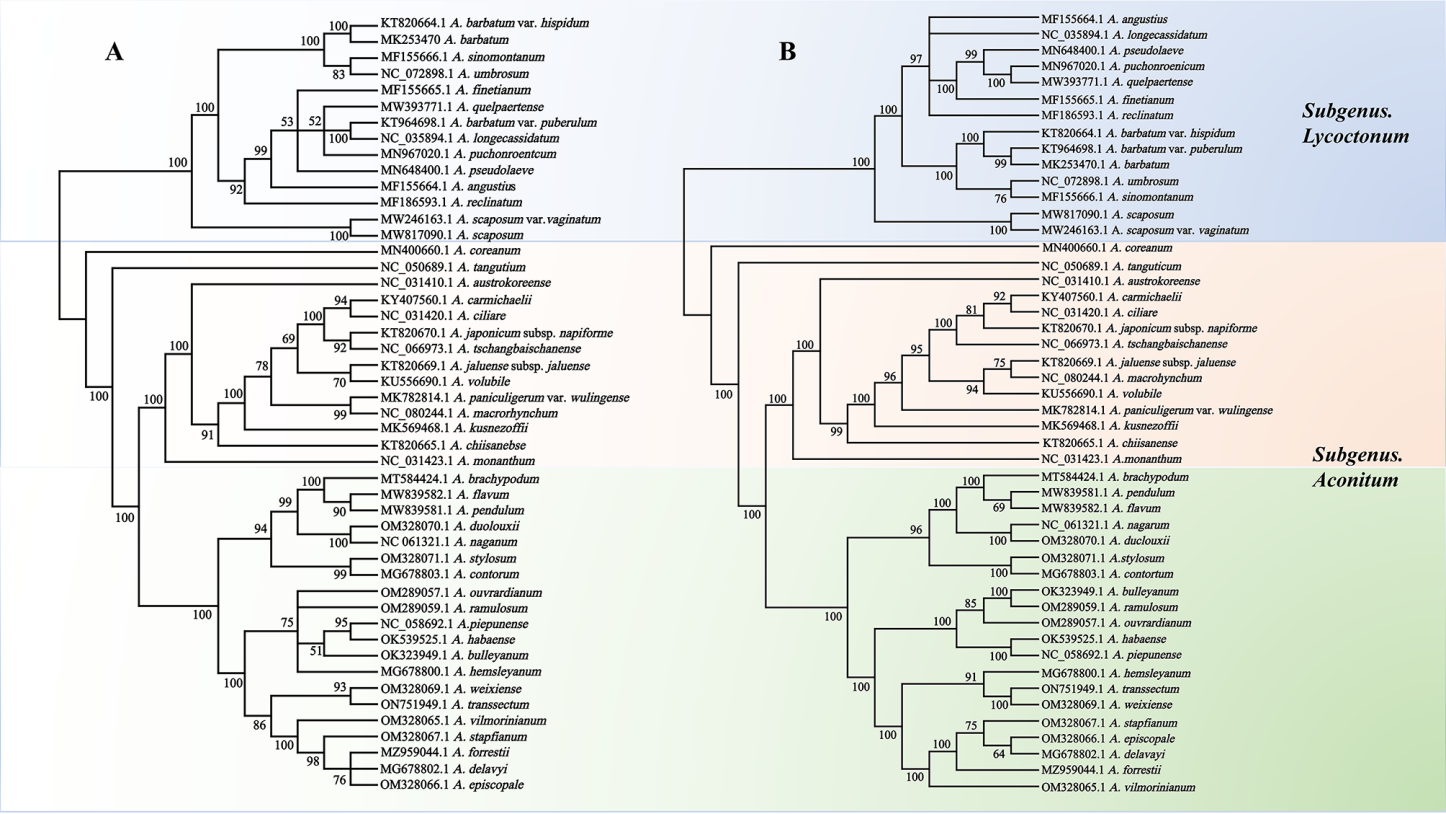
48 species of *Aconitum* using CDSs sequence by the maximum likelihood method (A) ML tree constructed based on cp. genome B ML tree constructed based on PCGs

The ML tree was utilized to design phylogenetic trees based on PCGs and cp. genome from 48 species to further explore the relationship between *Aconitum* species during evolution. ML tree showed results with a high support rate detected on most nodes. Of the 48 species of the *Aconitum*, which are mainly divided into the Subgenus. *Aconitum* and Subgenus. *Lycoctonum* (Fig. 9). Among them, Subgenus. *Lycoctonum* includes 14 species, namely *A. barbatum* var. *hispidum*, *A. barbatum*, *A. sinomontanum*, *A. umbrosum*, *A. finetianum*, *A. quelpaertense*, *A. barbatum* var. puberulum, *A. longecassidatum*, *A. puchonroentcum*, *A. pseudolaeve*, *A. angustius*, *A. reclinatum*, *A. scaposum* var. *vaginatum*, *A. sacposum* and Subgenus. *Aconitum* mainly includes 34 species. Moreover, Subgenus. *Aconitum* is evolutionarily consistent in dendrograms constructed from different sequences, especially in the orange-colored section, which shows a high degree of similarity, and in the green-colored area, which is divided into two branches, and, in the first branch, the seven species have the same evolutionary pattern. In contrast, in the second branch, the evolutionary relationship seems different. The CDS-constructed dendrograms taught us that *A. stapfianum* is more advanced than *A. forrestii*. However, it is the opposite in the genome-constructed dendrograms; moreover, *A. hemsleyanum* also shows different evolutionary patterns in different dendrograms. The ML phylogenetic tree based on PCGs and cp. genomes shows that most species are very similar at the level of evolutionary relationships, especially in the Subgenus *Aconitum*, suggesting that site-specific mutational features of coding sequences play an important role in biological evolution.

## Methods

### Genomes and sequence data Collection

The datasets of PCGs of 48 *Aconitum* species were downloaded from the National Center for Biotechnology Information’s (NCBI; http://www.ncbi.nlm.nih.gov/) genome sequence databases, and the accession numbers are listed in Supplementary Table 1. The Coding Sequences (CDS) were screened from genome-wide data for further analysis to ensure the results’ precision; the CDS length should be ≥ 300 bp, and removing redundant records of CDSs for the same protein-coding gene should be done. Calculations were done for *Aconitum* species’ CDS numbers, codons’ base compositions at the first/second/third sites (GC_1_/GC_2_/GC_3_), and overall GC (GC_ALL_). The nucleobase content at the location of the third codon (A3, T3, C3, G3), the ENCs, and RSCU heatmaps for all synonymous codons were performed using the CodonW (http://codonw.sourceforge.Net) software.

### RSCU analysis

The RSCU analysis of 48 *Aconitum* species was clustered using the TBtools v1.0987663 software (https://github.com/CJ-Chen/TBtools/releases) for clustering with RSCU, hierarchical clustering, and Euclidean distance was utilized. The ratio of the observed usage frequency of one codon in a coding sequence to the expected usage frequency in the synonymous codon family is used to determine the relative codon usage frequency or RSCU. The codons were considered to have a solid positive with an RSCU value > 1 [21].

### Selection of optimal Codon

Synonymous codons with the highest frequency and maximum RSCU value could be identified from the RSCU analysis [22]. The sequences were ordered according to their ENC ratio, where 10% of all genes were structured into high and low-expression gene data sets with the highest and lowest ENC values, respectively. RSCU analysis shows high-expression codons that appear significantly and more frequently expressed than low-expression genes.

RSCU measures the distinction between high and low expression. Finally, the optimal codon for cp. genomes was identified as satisfying RSCU > 1 and ∆RSCU ≥ 0.08 values [23].

### Correlation analysis and ENC-Plot analysis

SPSS tool was used for correlation analysis between different parameters, and the heatmap analysis was performed using the online Chiplot software (https://www.chiplot.online). A random selection of ENC value can be used for the degree of deviation of condos, which signifies the imbalance degree of synonymous codon usage. A scatter plot of the ENC values against GC_3_ values was represented to estimate the codon bias. According to Eq. 1 [24], expected ENC values were calculated, where it means and makes a standard line. The point is on or near the theoretical value distribution on the graph, showing that mutation is the primary factor. At the same time, the plot is distant from each point when the selection action changes the skewness. Moreover, Eq. 2 shows the difference between the observed ENC and actual values [25, 26].

1 $$\text{E}\text{N}\text{C}=2+\text{s}+\frac{29}{{\text{s}}^{2}+{\left(1-\text{s}\right)}^{2}}$$

2 $$\text{E}\text{N}\text{C}\text{r}\text{a}\text{t}\text{i}\text{o}=\frac{\left(ENCexp - ENCobs \right)}{ENCexp}$$

### PR2-bias plot analysis

PR2 was drawn by G3/(G3 + C3) as abscissa and A3/(A3 + T3) as ordinate in Fig. [27]. Based on this, the distribution of the four bases at the third position codon is determined. Two lines were drawn at X = 0.5 and Y = 0.5. The bases A and T and G and C are equal, as indicated by the intersection points. There is a distinction in using bases characterized by the distribution of other attributes.

### Comparative analysis of sequence differences

We utilized MAFFT v. 7 129 [28] software to align the chloroplast genome sequences of 48 *Aconitum* plants. We then performed sliding window analysis on nucleotide variability (Pi) in the chloroplast genome using DnaSP v.7.0.26 [29]. The step size was 200 bp, and the window length was 600 bp. Statistical analysis was conducted using Excel. To format and convert the annotations of cp. genome sequences, we used a coding program and the online software mVISTA [30]. PCR primer design for highly variable loci using NCBI Primer-BLAST (https://www.ncbi.nlm.nih.gov/tools/primer-blast/index.cgi?LINK_LOC=BlastHome).

### Construction of ML phylogenetic tree based on PCGs

The ML phylogenetic tree was designed using 48 PCG sequences of *Aconitum* species. MAFFT v. seven programs [28] was used to perform alienation of the PCG sequences. The RaxML version was used to collect data for the ML bootstrap analysis using GTR + R6 and 1000 bootstrap [31].

## Discussion

This study aimed to examine the codon usage patterns of 48 *Aconitum* species. Over time, *Aconitum* plants have developed unique codon usage patterns to adapt to various factors, including natural selection, mutational pressure, and gene level versus evolution. The study also analyzed the sources of variation in codon usage. The findings regarding codon preference usage patterns and optimal codons could help optimize heterologous genes and genetic evolution.

The genome’s gene variation can be conveyed and analyzed through codons, ultimately determining the encoded protein [32]. Notably, the third base of synonymous codons is the primary distinguishing factor, and GC_3_ is frequently employed to assess codon preference since the third position of synonymous codons is subject to selective pressure [33]. Previous studies have revealed a significant divergence in GC_3_ content between monocots and dicots. Specifically, monocots exhibit GC_3_ values higher than 50% and a preference for C/G codon usage, while the opposite is true for dicots [26]. Our study observed a trend of increasing GC content from GC_3_ to GC_2_ to GC_1_, with all three positions exhibiting a GC content of less than 50%.

Interestingly, the *Aconitum* genus codon prefers an A/T base, clarifying the base composition of *Aconitum* plants. Furthermore, the RSCU heatmap analysis revealed that 48 *Aconitum* species prefer A/T codon usage. This finding is consistent with previous studies on dicotyledonous plants such as *Panicum miliaceum* L [34], showing that closely related higher plants have some similarity in codon preference usage patterns and reinforces the results obtained for *Aconitum*.

Plants and genes exhibit a diverse range of codon usage patterns. Over time, organisms have evolved distinct codon usage patterns to better adapt to their environment [35]. Synonymous codon bias can be influenced by various factors, with natural selection and mutational pressure being the primary drivers [36]. Our analysis revealed that the codon usage patterns of *Aconitum* were impacted by natural selection, as demonstrated by the ENC-plot analysis. These findings were consistent with the codon usage patterns observed in *Euphorbiaceae* [37] and *Poaceae* [38]. Based on PR2-plot and correlation analysis, reports also mention similar codon usage patterns in *Asteraceae* [39]. However, this conclusion contradicts the idea that mango has a relatively balanced effect of mutations and natural selection on codon preference [40]. This suggests that the primary factors influencing codon preference vary across different species.

We can predict and manipulate gene expression levels in an organism’s genome by identifying the optimal codons. This knowledge can be applied in various fields, including biotechnology and medicine. Furthermore, studying codon usage preference can provide insights into the evolutionary history of a species and its genetic code. Therefore, investigating the characteristics of codon usage preference is crucial for understanding the fundamental processes of gene expression and evolution. Research has demonstrated that the selective use of synonymous codons can influence gene expression. Optimal codons have been found to enhance the efficient and accurate translation of genes, leading to higher expression levels [48]. In 48 *Aconitum* species, we have identified 7–16 optimal codons for each species. These codons include CAU, UGA, and GGA, commonly used across the *Aconitum* genus. The more biased a species is towards using these codons, the higher its gene expression level tends to be [41]. As a result, the leaf-green genome of species that use these optimal codons may have higher expression levels. Interestingly, there is a higher prevalence of A/T ending codon, consistent with the findings of codon bias studies in *Gymnostemma*. [42].

SNP markers are commonly utilized in crop genetic background selection and molecular-assisted breeding due to their accuracy and high reproducibility [43, 44]. We screened the various sites of *Aconitum* species by nucleic acid polymorphisms, including *psbT-psbN*, *trnA-UGC*, *trnI-GAU*, *psbD*, *petB* sequences,, and these regions could be used as novel candidate fragments to identify *Aconitum* species. However, the choice of core barcodes also requires data support from more chloroplast genome sequences of *Aconitum* plants, an issue that should be addressed in future studies. Further, a greater genetic distance leads to a more distinct bias in codon usage. This finding suggests that codon usage bias can be valuable for determining evolutionary relationships between species. These results provide insight into the complex interplay between genetic and environmental factors in shaping the evolution of species [45]. In this study, the phylogenetic tree based on PCGs showed that species of different subgenera formed different branches, consistent with the ml tree results based on the whole genome composition of chloroplasts reported previously [46]. However, other evolutionary relationships were shown in the subgenus *Aconitum*, which may be because the genomes contain more sequences from non-coding regions and protein codes prone to mutation. Thus there are some relative differences which affected the accuracy of the results of intergenomic distance calculations. In addition, this paper even divided the subgen. *Aconitum* into two branches again showed similar patterns between phylogenetic trees constructed from different sequences, clarifying the importance of protein-coding columns in inheritance and conserved phenotypes.

This study thoroughly evaluates codon usage patterns in 48 *Aconitum* species. This knowledge will improve our understanding of codons for analyzing the cp. genome and optimizing gene expression. Ultimately, this will aid in developing *Aconitum* or other higher plant species providing a theoretical foundation for constructing stable and efficient gene expression constructs.

## Conclusion

In this study, we analyzed codon preferences of 48 species of *Aconitum* and showed that A/T-rich codons dominate codons coding for proteins.RSCU analyses further revealed that all 48 species preferred codons ending in A/T. In addition, we observed differences in optimal codon usage within the chloroplast genome. At the same time, we delved into codon usage patterns. Our analysis suggests that natural selection is essential in causing codon usage bias, while pressure selection and gene level also impact codon bias. In addition, the phylogenetic tree results indicate that the Subgen. *Aconitum* and Subgen. *Lycoctonum* forms distinct branches. We also found that using PCG to analyze species evolution is reliable.

### Electronic supplementary material

Below is the link to the electronic supplementary material.

Supplementary Material 1

Supplementary Material 2

Supplementary Material 3

Supplementary Material 4

Supplementary Material 5

Supplementary Material 6

## Data Availability

The datasets of the other 48 plants analysis are available in the NCBI database (http://www.ncbi.nlm.nih.gov/).

## List of Abbreviations

CUB: Codon usage bias
PCGs: Protein-coding sequences
RSCU: Relative synonymous codon usage
ENC: The adequate number of codons
PR2: The parity rule 2
ML: Maximum Likelihood
cp: Chloroplast
TCM: Traditional Chinese medicine
CDS: Coding Sequences
