# Supplementary material for: Analysis of codon usage patterns in 48 Aconitum species
Source: BMC Genomics. 2023 Nov 22;24:703. doi: 10.1186/s12864-023-09650-5 (PMC10664653; doi:10.1186/s12864-023-09650-5)
Supplement: Supplementary file 1 — Supplementary Material 1 [file 12864_2023_9650_MOESM1_ESM.docx]

| **Taxon** | **Accession number in the NCBI database** | **CDSs number**  **(after filting)** | **Taxon** | **Accession number in the NCBI database** | **CDSs number**  **(after filting)** |
| --- | --- | --- | --- | --- | --- |
| ***Aconitum angustius*** | **MF155664** | **53** | ***Aconitum kusnezoffii*** | **MK569468.1** | **53** |
| ***Aconitum austrokoreense*** | **NC_031410.1** | **54** | ***Aconitum longecassidatum*** | **NC_035894.1** | **53** |
| ***Aconitum barbatum*** | **MK253470** | **53** | ***Aconitum monanthum*** | **NC_031423.1** | **53** |
| ***Aconitum barbatum* var.hispidum** | **KT820664.1** | **54** | ***Aconitum nagarum*** | **NC_061321.1** | **53** |
| ***Aconitum barbatum* var.puberulum** | **KT964698** | **53** | ***Aconitum ouvrardianum*** | **OM289057.1** | **53** |
| ***Aconitum brachypodum*** | **MT584424.1** | **53** | ***Aconitum pendulum*** | **MW839581.1** | **53** |
| ***Aconitum bulleyanum*** | **OK323949.1** | **53** | ***Aconitum piepunense*** | **NC_058692.1** | **53** |
| ***Aconitum carmichaelii*** | **KY407560.1** | **53** | ***Aconitum pseudolaeve*** | **MN648400.1** | **53** |
| ***Aconitum chiisanense*** | **KT820665.1** | **53** | ***Aconitum puchonroenicum*** | **MN967020.1** | **53** |
| ***Aconitum ciliare*** | **NC_031420.1** | **53** | ***Aconitum quelpaertense*** | **MW393771.1** | **53** |
| ***Aconitum contortum*** | **MG678803.1** | **53** | ***Aconitum ramulosum*** | **OM289059.1** | **53** |
| ***Aconitum coreanum*** | **MN400660.1** | **53** | ***Aconitum reclinatum*** | **MF186593.1** | **52** |
| ***Aconitum delavayi*** | **MG678802.1** | **53** | ***Aconitum scaposum*** | **MW817090.1** | **53** |
| ***Aconitum duclouxii*** | **OM328070.1** | **53** | ***Aconitum scaposum* var. vaginatum** | **MW246163.1** | **52** |
| ***Aconitum episcopale*** | **OM328066.1** | **53** | ***Aconitum sinomontanum*** | **MF155666.1** | **53** |
| ***Aconitum finetianum*** | **MF155665.1** | **53** | ***Aconitum stapfianum*** | **OM328067.1** | **53** |
| ***Aconitum flavum*** | **MW839582.1** | **53** | ***Aconitum stylosum*** | **OM328071.1** | **53** |
| ***Aconitum hemsleyanum*** | **MG678800.1** | **53** | ***Aconitum tanguticum*** | **NC_050689.1** | **53** |
| ***Aconitum forrestii*** | **MZ959044** | **53** | ***Aconitum vilmorinianum*** | **OM328065.1** | **53** |
| ***Aconitum jaluense* subsp. jaluense** | **KT820669.1** | **53** | ***Aconitum weixiense*** | **OM328069.1** | **53** |
| ***Aconitum japonicum* subsp. napiforme** | **KT820670.1** | **53** | **KU556690.1** | **Aconitum volubile** |  |
| **Aconitum habaense** | **Ok539525.1** |  | **NC_066973** | **Aconitum tschangbaischanense** |  |
| **Aconitum transsectum** | **ON751949.1** |  | **NC_072898.1** | **Aconitum umbrosum** |  |
| **Aconitum macrohynchum** | **NC_080244** |  | **MK782814.1** | **Aconitum paniculigerum var. wulingense** |  |

Supplementary Table 1 Accession number in the NCBI database and the number of CDS in 41 *Aconitum* species
