# Supplementary material for: Analysis of codon usage patterns in 48 Aconitum species
Source: BMC Genomics. 2023 Nov 22;24:703. doi: 10.1186/s12864-023-09650-5 (PMC10664653; doi:10.1186/s12864-023-09650-5)
Supplement: Supplementary file 5 — Supplementary Material 5 [file 12864_2023_9650_MOESM5_ESM.docx]

|  | GC_ALL_ | GC_1_ | GC_2_ | GC_3_ | GC_3s_ | A_3_ | T_3_ | C_3_ | G_3_ | ENC | CAI | CBI |
| --- | --- | --- | --- | --- | --- | --- | --- | --- | --- | --- | --- | --- |
| GCALL | 1 | 0.801** | 0.75** | 0.538** | 0.5113** | -0.339* | -0.33* | 0.487** | -0.03 | 0.192 | 0.401** | 0.545** |
| GC1 | 0.801 | 1 | 0.359** | 0.242 | 0.336* | -0.212 | -0.213 | 0.305* | -0.033 | 0.074 | 0.368** | 0.442** |
| GC2 | 0.75 | 0.359 | 1 | 0.12 | 0.067 | -0.057 | -0.334* | 0.182 | -0.266 | -0.006 | 0.072 | 0.322* |
| GC3 | 0.538 | 0.242 | 0.12 | 1 | 0.865** | -0.567** | -0.127 | 0.664** | 0.361** | 0.448** | 0.486** | 0.414** |
| GC3s | 0.5113 | 0.336 | 0.067 | 0.865 | 1 | -0.58** | -0.211 | 0.652** | 0.532** | 0.504** | 0.354** | 0.401** |
| A3 | -0.339 | -0.212 | -0.057 | -0.567 | -0.58 | 1 | -0.472** | -0.328* | -0.252 | -0.156 | -0.498** | -0.384** |
| T3 | -0.33 | -0.213 | -0.334 | -0.127 | -0.211 | -0.472 | 1 | -0.29* | 0.121 | -0.119 | 0.265 | -0.145 |
| C3 | 0.487 | 0.305 | 0.182 | 0.664 | 0.652 | -0.328 | -0.29 | 1 | -0.239 | 0.351** | 0.617** | 0.681** |
| G3 | -0.03 | -0.033 | -0.266 | 0.361 | 0.532 | -0.252 | 0.121 | -0.239 | 1 | 0.383** | -0.235 | -0.331* |
| ENC | 0.192 | 0.074 | -0.006 | 0.448 | 0.504 | -0.156 | -0.119 | 0.351 | 0.383 | 1 | 0.026 | -0.041 |
| CAI | 0.401 | 0.368 | 0.072 | 0.486 | 0.354 | -0.498 | 0.265 | 0.617 | -0.235 | 0.026 | 1 | 0.752** |
| CBI | 0.545 | 0.442 | 0.322 | 0.414 | 0.401 | -0.384 | -0.145 | 0.681 | -0.331 | -0.041 | 0.752 | 1 |

**Supplementary Table 5 Correlation analysis in 41 *Aconitum***

1. *angustius*

|  | GC_ALL_ | GC_1_ | GC_2_ | GC_3_ | GC_3s_ | A_3_ | T_3_ | C_3_ | G_3_ | ENC | CAI | CBI |
| --- | --- | --- | --- | --- | --- | --- | --- | --- | --- | --- | --- | --- |
| GCALL | 1 | 0.771** | 0.797** | 0.525** | 0.521** | -0.398** | -0.461** | 0.465** | -0.029 | 0.276* | 0.277* | 0.409** |
| GC1 | 0.771 | 1 | 0.402** | 0.18 | 0.252 | -0.153 | -0.371** | 0.38** | -0.213 | 0.236 | 0.381** | 0.475** |
| GC2 | 0.797 | 0.402 | 1 | 0.169 | 0.154 | -0.207 | -0.426** | 0.133 | -0.184 | -0.027 | -0.025 | 0.179 |
| GC3 | 0.525 | 0.18 | 0.169 | 1 | 0.899** | -0.621** | -0.112 | 0.577** | 0.522** | 0.47** | 0.269* | 0.172 |
| GC3s | 0.521 | 0.252 | 0.154 | 0.899 | 1 | -0.643 | -0.152 | 0.653** | 0.557** | 0.508** | 0.337* | 0.276* |
| A3 | -0.398 | -0.153 | -0.207 | -0.621 | -0.643 | 1 | -0.44 | -0.402** | -0.282* | -0.189 | -0.491** | -0.303* |
| T3 | -0.461 | -0.371 | -0.426 | -0.112 | -0.152 | -0.44 | 1 | -0.224 | 0.128 | -0.095 | 0.288* | -0.121 |
| C3 | 0.465 | 0.38 | 0.133 | 0.577 | 0.653 | -0.402 | -0.224 | 1 | -0.211 | 0.392** | 0.559** | 0.506** |
| G3 | -0.029 | -0.213 | -0.184 | 0.522 | 0.557 | -0.282 | 0.128 | -0.211 | 1 | 0.355** | -0.205 | -0.310* |
| ENC | 0.276 | 0.236 | -0.027 | 0.47 | 0.508 | -0.189 | -0.095 | 0.392 | 0.355 | 1 | 0.048 | -0.07 |
| CAI | 0.277 | 0.381 | -0.025 | 0.269 | 0.337 | -0.491 | 0.288 | 0.559 | -0.205 | 0.048 | 1 | 0.731** |
| CBI | 0.409 | 0.475 | 0.179 | 0.172 | 0.276 | -0.303 | -0.121 | 0.506 | -0.310 | -0.07 | 0.731 | 1 |

*A. austrokoreense*

*A. barbatum*

|  | GC_ALL_ | GC_1_ | GC_2_ | GC_3_ | GC_3s_ | A_3_ | T_3_ | C_3_ | G_3_ | ENC | CAI | CBI |
| --- | --- | --- | --- | --- | --- | --- | --- | --- | --- | --- | --- | --- |
| GCALL | 1 | 0.791** | 0.758** | 0.533** | 0.492** | -0.322* | -0.314* | 0.447** | -0.002 | 0.251 | 0.391** | 0.526** |
| GC1 | 0.791 | 1 | 0.356** | 0.219 | 0.289* | -0.212 | -0.181 | 0.261 | -0.049 | 0.08 | 0.375** | 0.453** |
| GC2 | 0.758 | 0.356 | 1 | 0.135 | 0.075 | -0.035 | -0.345* | 0.159 | -0.223 | 0.09 | 0.055 | 0.301* |
| GC3 | 0.533 | 0.219 | 0.135 | 1 | 0.866** | -0.553** | -0.107 | 0.652** | 0.405** | 0.467** | 0.473** | 0.374** |
| GC3s | 0.492 | 0.289 | 0.075 | 0.866 | 1 | -0.587** | -0.182 | 0.639** | 0.563** | 0.563** | 0.34* | 0.355** |
| A3 | -0.322 | -0.212 | -0.035 | -0.553 | -0.587 | 1 | -0.496** | -0.329* | -0.28* | -0.164 | -0.499** | -0.362** |
| T3 | -0.314 | -0.181 | -0.345 | -0.107 | -0.182 | -0.496 | 1 | -0.24 | 0.101 | -0.162 | 0.293 | -0.113 |
| C3 | 0.447 | 0.261 | 0.159 | 0.652 | 0.639 | -0.329 | -0.24 | 1 | -0.219 | 0.368** | 0.613** | 0.661** |
| G3 | -0.002 | -0.049 | -0.223 | 0.405 | 0.563 | -0.28 | 0.101 | -0.219 | 1 | 0.388** | -0.23 | -0.346* |
| ENC | 0.251 | 0.08 | 0.09 | 0.467 | 0.563 | -0.164 | -0.162 | 0.368 | 0.388 | 1 | 0.013 | -0.033 |
| CAI | 0.391 | 0.375 | 0.055 | 0.473 | 0.34 | -0.499 | 0.293 | 0.613 | -0.23 | 0.013 | 1 | 0.75** |
| CBI | 0.526 | 0.453 | 0.301 | 0.374 | 0.355 | -0.362 | -0.113 | 0.661 | -0.346 | -0.033 | 0.75 | 1 |

|  | GC_ALL_ | GC_1_ | GC_2_ | GC_3_ | GC_3s_ | A_3_ | T_3_ | C_3_ | G_3_ | ENC | CAI | CBI |
| --- | --- | --- | --- | --- | --- | --- | --- | --- | --- | --- | --- | --- |
| GCALL | 1 | 0.784** | 0.765** | 0.548** | 0.508** | -0.396** | -0.187 | 0.411** | 0.067 | 0.327* | 0.407** | 0.469** |
| GC1 | 0.784 | 1 | 0.353** | 0.221 | 0.275* | -0.176 | -0.205 | 0.295* | -0.083 | 0.107 | 0.434** | 0.46** |
| GC2 | 0.765 | 0.353 | 1 | 0.164 | 0.119 | -0.175 | -0.143 | 0.099 | -0.101 | 0.185 | 0.063 | 0.211 |
| GC3 | 0.548 | 0.221 | 0.164 | 1 | 0.871** | -0.614 | -0.0115 | 0.593** | 0.471** | 0.501** | 0.426** | 0.341* |
| GC3s | 0.508 | 0.275 | 0.119 | 0.871 | 1 | -0.581** | -0.201 | 0.634** | 0.569** | 0.534** | 0.339* | 0.353** |
| A3 | -0.396 | -0.176 | -0.175 | -0.614 | -0.581 | 1 | -0.482** | -0.328* | -0.272* | -0.169 | -0.5** | -0.362** |
| T3 | -0.187 | -0.205 | -0.143 | -0.0115 | -0.201 | -0.482 | 1 | -0.232 | 0.071 | -0.164 | 0.279* | -0.114 |
| C3 | 0.411 | 0.295 | 0.099 | 0.593 | 0.634 | -0.328 | -0.232 | 1 | -0.271 | 0.37** | 0.608** | 0.653** |
| G3 | 0.067 | -0.083 | -0.101 | 0.471 | 0.569 | -0.272 | 0.071 | -0.271 | 1 | 0.384** | -0.227 | -0.338* |
| ENC | 0.327 | 0.107 | 0.185 | 0.501 | 0.534 | -0.169 | -0.164 | 0.37 | 0.384 | 1 | 0.013 | -0.023 |
| CAI | 0.407 | 0.434 | 0.063 | 0.426 | 0.339 | -0.5 | 0.279 | 0.608 | -0.227 | 0.013 | 1 | 0.753** |
| CBI | 0.469 | 0.46 | 0.211 | 0.341 | 0.353 | -0.362 | -0.114 | 0.653 | -0.338 | -0.023 | 0.753 | 1 |

*A. barbatum* var. *hispidum*

*A. barbatum* var. *puberulum*

|  | GC_ALL_ | GC_1_ | GC_2_ | GC_3_ | GC_3s_ | A_3_ | T_3_ | C_3_ | G_3_ | ENC | CAI | CBI |
| --- | --- | --- | --- | --- | --- | --- | --- | --- | --- | --- | --- | --- |
| GCALL | 1 | 0.795** | 0.75** | 0.505** | 0.452** | -0.382* | -0.278* | 0.458** | -0.004 | 0.185 | 0.401** | 0.519** |
| GC1 | 0.795 | 1 | 0.383** | 0.168 | 0.239 | -0.19 | -0.14 | 0.248 | -0.049 | 0.018 | 0.388** | 0.438** |
| GC2 | 0.75 | 0.383 | 1 | 0.079 | 0.084 | -0.097 | -0.279* | 0.173 | -0.2 | 0.015 | 0.039 | 0.274* |
| GC3 | 0.505 | 0.168 | 0.079 | 1 | 0.752** | -0.47** | -0.158 | 0.63** | 0.333* | 0.449** | 0.463** | 0.383** |
| GC3s | 0.452 | 0.239 | 0.084 | 0.752 | 1 | -0.576** | -0.191 | 0.66** | 0.622** | 0.516** | 0.393** | 0.391** |
| A3 | -0.382 | -0.19 | -0.097 | -0.47 | -0.576 | 1 | -0.499** | -0.319* | -0.3* | -0.118 | -0.517** | -0.39** |
| T3 | -0.278 | -0.14 | -0.279 | -0.158 | -0.191 | -0.499 | 1 | -0.318* | 0.1 | -0.221 | 0.262 | -0.11 |
| C3 | 0.458 | 0.248 | 0.173 | 0.63 | 0.66 | -0.319 | -0.318 | 1 | -0.132 | 0.338* | 0.609** | 0.681** |
| G3 | -0.004 | -0.049 | -0.2 | 0.333 | 0.622 | -0.3 | 0.1 | -0.132 | 1 | 0.412** | -0.12 | -0.254 |
| ENC | 0.185 | 0.018 | 0.015 | 0.449 | 0.516 | -0.118 | -0.221 | 0.338 | 0.412 | 1 | -0.042 | -0.072 |
| CAI | 0.401 | 0.388 | 0.039 | 0.463 | 0.393 | -0.517 | 0.262 | 0.609 | -0.12 | -0.042 | 1 | 0.773** |
| CBI | 0.519 | 0.438 | 0.274 | 0.383 | 0.391 | -0.39 | -0.11 | 0.681 | -0.254 | -0.072 | 0.773 | 1 |

|  | GC_ALL_ | GC_1_ | GC_2_ | GC_3_ | GC_3s_ | A_3_ | T_3_ | C_3_ | G_3_ | ENC | CAI | CBI |
| --- | --- | --- | --- | --- | --- | --- | --- | --- | --- | --- | --- | --- |
| GCALL | 1 | 0.807** | 0.77** | 0.537** | 0.511** | -0.347* | -0.326* | 0.426** | 0.042 | 0.259 | 0.379** | 0.468** |
| GC1 | 0.807 | 1 | 0.412** | 0.234 | 0.287* | -0.222 | -0.2 | 0.22 | -0.014 | 0.149 | -0.349* | 0.402** |
| GC2 | 0.77 | 0.412 | 1 | 0.125 | 0.099 | -0.131 | -0.383** | 0.175 | -0.214 | 0.04 | 0.068 | 0.29* |
| GC3 | 0.537 | 0.234 | 0.125 | 1 | 0.886** | -0.615** | -0.073 | 0.63** | 0.449** | 0.457** | 0.466** | 0.318* |
| GC3s | 0.511 | 0.287 | 0.099 | 0.886 | 1 | -0.614** | -0.165 | 0.66** | 0.556** | 0.509** | 0.352** | 0.305* |
| A3 | -0.347 | -0.222 | -0.131 | -0.615 | -0.614 | 1 | -0.48** | -0.345* | -0.309* | -0.181 | -0.537** | -0.343* |
| T3 | -0.326 | -0.2 | -0.383 | -0.073 | -0.165 | -0.48 | 1 | -0.273* | 0.159 | -0.119 | 0.289* | -0.131 |
| C3 | 0.426 | 0.22 | 0.175 | 0.63 | 0.66 | -0.345 | -0.273 | 1 | -0.204 | 0.387** | 0.589** | 0.598** |
| G3 | 0.042 | -0.014 | -0.214 | 0.449 | 0.556 | -0.309 | 0.159 | -0.204 | 1 | 0.343* | -0.214 | -0.359** |
| ENC | 0.259 | 0.149 | 0.04 | 0.457 | 0.509 | -0.181 | -0.119 | 0.387 | 0.343 | 1 | 0.088 | -0.016 |
| CAI | 0.379 | -0.349 | 0.068 | 0.466 | 0.352 | -0.537 | 0.289 | 0.589 | -0.214 | 0.088 | 1 | 0.727** |
| CBI | 0.468 | 0.402 | 0.29 | 0.318 | 0.305 | -0.343 | -0.131 | 0.598 | -0.359 | -0.016 | 0.727 | 1 |

*A. brachypodum*

*A.bulleyanum*

|  | GC_ALL_ | GC_1_ | GC_2_ | GC_3_ | GC_3s_ | A_3_ | T_3_ | C_3_ | G_3_ | ENC | CAI | CBI |
| --- | --- | --- | --- | --- | --- | --- | --- | --- | --- | --- | --- | --- |
| GCALL | 1 | 0.815** | 0.778** | 0.555** | 0.532** | -0.363** | -0.345* | 0.445** | 0.025 | 0.212 | 0.399** | 0.481** |
| GC1 | 0.815 | 1 | 0.431** | 0.252 | 0.314* | -0.242 | -0.214 | 0.249 | -0.021 | 0.125 | 0.358** | 0.427** |
| GC2 | 0.778 | 0.431 | 1 | 0.158 | 0.13 | -0.035 | -0.418** | 0.193 | -0.2 | 0.035 | 0.092 | 0.298* |
| GC3 | 0.555 | 0.252 | 0.158 | 1 | 0.883** | -0.64** | -0.075 | 0.636** | 0.393** | 0.376** | 0.489** | 0.325* |
| GC3s | 0.532 | 0.314 | 0.13 | 0.883 | 1 | -0.633** | -0.161 | 0.656** | 0.518** | 0.413** | 0.374** | 0.302* |
| A3 | -0.363 | -0.242 | -0.035 | -0.64 | -0.633 | 1 | -0.447** | -0.374** | -0.275* | -0.123 | -0.542** | -0.345* |
| T3 | -0.345 | -0.214 | -0.418 | -0.075 | -0.161 | -0.447 | 1 | -0.273* | 0.185 | -0.064 | 0.251 | -0.163 |
| C3 | 0.445 | 0.249 | 0.193 | 0.636 | 0.656 | -0.374 | -0.273 | 1 | -0.252 | 0.327* | 0.629** | 0.617** |
| G3 | 0.025 | -0.021 | -0.2 | 0.393 | 0.518 | -0.275 | 0.185 | -0.252 | 1 | 0.288* | -0.248 | -0.407** |
| ENC | 0.212 | 0.125 | 0.035 | 0.376 | 0.413 | -0.123 | -0.064 | 0.327 | 0.288 | 1 | 0.059 | -0.042 |
| CAI | 0.399 | 0.358 | 0.092 | 0.489 | 0.374 | -0.542 | 0.251 | 0.629 | -0.248 | 0.059 | 1 | 0.738** |
| CBI | 0.481 | 0.427 | 0.298 | 0.325 | 0.302 | -0.345 | -0.163 | 0.617 | -0.407 | -0.042 | 0.738 | 1 |

|  | GC_ALL_ | GC_1_ | GC_2_ | GC_3_ | GC_3s_ | A_3_ | T_3_ | C_3_ | G_3_ | ENC | CAI | CBI |
| --- | --- | --- | --- | --- | --- | --- | --- | --- | --- | --- | --- | --- |
| GCALL | 1 | 0.927** | 0.887** | 0.802** | 0.107 | -0.131 | -0.161 | -0.234 | -0.194 | -0.094 | 0.28* | 0.181 |
| GC1 | 0.927 | 1 | 0.727** | 0.66** | 0.049 | -0.014 | -0.12 | 0.141 | -0.177 | -0.131 | 0.279* | 0.192 |
| GC2 | 0.887 | 0.727 | 1 | 0.552** | -0.097 | 0.127 | -0.257 | 0.117 | -0.351** | 0.168 | 0.11 | 0.147 |
| GC3 | 0.802 | 0.66 | 0.552 | 1 | 0.439** | -0.271* | -0.013 | 0.437** | 0.097 | 0.115 | 0.392** | 0.128 |
| GC3s | 0.107 | 0.049 | -0.097 | 0.439 | 1 | -0.627** | -0.138 | 0.663** | 0.542** | 0.512** | 0.331 | 0.293* |
| A3 | -0.131 | -0.014 | 0.127 | -0.271 | -0.627 | 1 | -0.483** | -0.352** | -0.313* | -0.171 | -0.501** | -0.319* |
| T3 | -0.161 | -0.12 | -0.257 | -0.013 | -0.138 | -0.483 | 1 | -0.253 | 0.184 | -0.086 | 0.291* | -0.143 |
| C3 | -0.234 | 0.141 | 0.117 | 0.437 | 0.663 | -0.352 | -0.253 | 1 | -0.215 | 0.374** | 0.573** | 0.585** |
| G3 | -0.194 | -0.177 | -0.351 | 0.097 | 0.542 | -0.313 | 0.184 | -0.215 | 1 | 0.38** | -0.229 | -0.376** |
| ENC | -0.094 | -0.131 | 0.168 | 0.115 | 0.512 | -0.171 | -0.086 | 0.374 | 0.38 | 1 | 0.021 | -0.078 |
| CAI | 0.28 | 0.279 | 0.11 | 0.392 | 0.331 | -0.501 | 0.291 | 0.573 | -0.229 | 0.021 | 1 | 0.721** |
| CBI | 0.181 | 0.192 | 0.147 | 0.128 | 0.293 | -0.319 | -0.143 | 0.585 | -0.376 | -0.078 | 0.721 | 1 |

1. *carmichaelii*

*A.chiisanense*

|  | GC_ALL_ | GC_1_ | GC_2_ | GC_3_ | GC_3s_ | A_3_ | T_3_ | C_3_ | G_3_ | ENC | CAI | CBI |
| --- | --- | --- | --- | --- | --- | --- | --- | --- | --- | --- | --- | --- |
| GCALL | 1 | 0.813** | 0.732** | 0.505** | 0.356** | -0.315* | -0.217 | 0.287* | -0.035 | 0.121 | 0.328* | 0.422** |
| GC1 | 0.813 | 1 | 0.378** | 0.21 | 0.078 | -0.052 | -0.184 | 0.207 | -0.224 | 0.113 | 0.36** | 0.405 |
| GC2 | 0.732 | 0.378 | 1 | 0.047 | -0.003 | -0.13 | -0.259 | -0.04 | -0.192 | -0.189 | -0.001 | 0.233 |
| GC3 | 0.505 | 0.21 | 0.047 | 1 | 0.858** | -0.601** | 0.039 | 0.53** | 0.501** | 0.423** | 0.362** | 0.228 |
| GC3s | 0.356 | 0.078 | -0.003 | 0.858 | 1 | -0.645** | -0.097 | 0.638** | 0.554** | 0.521** | 0.323* | 0.259 |
| A3 | -0.315 | -0.052 | -0.13 | -0.691 | -0.645 | 1 | -0.474** | -0.356** | -0.312* | -0.176 | -0.498** | -0.325* |
| T3 | -0.217 | -0.184 | -0.259 | 0.039 | -0.097 | -0.474 | 1 | -0.207 | 0.184 | -0.088 | 0.306* | -0.117 |
| C3 | 0.287 | 0.207 | -0.04 | 0.53 | 0.638 | -0.356 | -0.207 | 1 | -0.23 | 0.359** | 0.569** | 0.56** |
| G3 | -0.035 | -0.224 | -0.192 | 0.501 | 0.554 | -0.312 | 0.184 | -0.23 | 1 | 0.392** | -0.228 | -0.384** |
| ENC | 0.121 | 0.113 | -0.189 | 0.423 | 0.521 | -0.176 | -0.088 | 0.359 | 0.392 | 1 | 0.03 | -0.079 |
| CAI | 0.328 | 0.36 | -0.001 | 0.362 | 0.323 | -0.498 | 0.306 | 0.569 | -0.228 | 0.03 | 1 | 0.726** |
| CBI | 0.422 | 0.405 | 0.233 | 0.228 | 0.259 | -0.325 | -0.117 | 0.56 | -0.384 | -0.079 | 0.726 | 1 |

*A.ciliare*

|  | GC_ALL_ | GC_1_ | GC_2_ | GC_3_ | GC_3s_ | A_3_ | T_3_ | C_3_ | G_3_ | ENC | CAI | CBI |
| --- | --- | --- | --- | --- | --- | --- | --- | --- | --- | --- | --- | --- |
| GCALL | 1 | 0.807** | 0.773** | 0.522** | 0.498** | -0.333* | -0.333* | 0.444** | -0.005 | 0.222 | 0.376** | 0.479** |
| GC1 | 0.807 | 1 | 0.416** | 0.218 | 0.29* | -0.213 | -0.217 | 0.235 | -0.033 | 0.074 | 0.341* | 0.399** |
| GC2 | 0.773 | 0.416 | 1 | 0.112 | 0.077 | -0.016 | -0.387** | 0.185 | -0.259 | 0.028 | 0.075 | 0.314* |
| GC3 | 0.522 | 0.218 | 0.112 | 1 | 0.878** | -0.612** | -0.059 | 0.646** | 0.416** | 0.481** | 0.459** | 0.313* |
| GC3s | 0.498 | 0.29 | 0.077 | 0.878 | 1 | -0.624** | -0.141 | 0.662** | 0.545** | 0.511** | 0.328* | 0.290 * |
| A3 | -0.333 | -0.213 | -0.016 | -0.612 | -0.624 | 1 | -0.485** | -0.356** | -0.305* | -0.174 | -0.501** | -0.324* |
| T3 | -0.333 | -0.217 | -0.387 | -0.059 | -0.141 | -0.485 | 1 | 0.244 | 0.168 | -0.088 | 0.295* | -0.129 |
| C3 | 0.444 | 0.235 | 0.185 | 0.646 | 0.662 | -0.356 | 0.244 | 1 | -0.212 | 0.377** | 0.575** | 0.585** |
| G3 | -0.005 | -0.033 | -0.259 | 0.416 | 0.545 | -0.305 | 0.168 | -0.212 | 1 | 0.371** | -0.232 | -0.375** |
| ENC | 0.222 | 0.074 | 0.028 | 0.481 | 0.511 | -0.174 | -0.088 | 0.377 | 0.371 | 1 | 0.027 | -0.066 |
| CAI | 0.376 | 0.341 | 0.075 | 0.459 | 0.328 | -0.501 | 0.295 | 0.575 | -0.232 | 0.027 | 1 | 0.726** |
| CBI | 0.479 | 0.399 | 0.314 | 0.313 | 0.290 | -0.324 | -0.129 | 0.585 | -0.375 | -0.066 | 0.726 | 1 |

*A.contortum*

|  | GC_ALL_ | GC_1_ | GC_2_ | GC_3_ | GC_3s_ | A_3_ | T_3_ | C_3_ | G_3_ | ENC | CAI | CBI |
| --- | --- | --- | --- | --- | --- | --- | --- | --- | --- | --- | --- | --- |
| GCALL | 1 | 0.81** | 0.778** | 0.557** | 0.538** | -0.367** | -0.358** | 0.457** | 0.028 | 0.216 | 0.408** | 0.509** |
| GC1 | 0.81 | 1 | 0.426** | 0.245 | 0.311* | -0.231 | -0.224 | 0.245 | -0.014 | 0.121 | 0.362** | 0.431** |
| GC2 | 0.778 | 0.426 | 1 | 0.161 | 0.136 | -0.045 | -0.419** | 0.2 | -0.204 | 0.042 | 0.102 | 0.326* |
| GC3 | 0.557 | 0.245 | 0.161 | 1 | 0.89** | -0.645** | -0.092 | 0.66** | 0.394** | 0.38** | 0.492** | 0.358** |
| GC3s | 0.538 | 0.311 | 0.136 | 0.89 | 1 | -0.642** | -0.165 | 0.673** | 0.521** | 0.419** | 0.385** | 0.328* |
| A3 | -0.367 | -0.231 | -0.045 | -0.645 | -0.642 | 1 | -0.435** | -0.391** | -0.283* | -0.124 | -0.54** | -0.371** |
| T3 | -0.358 | -0.224 | -0.419 | -0.092 | -165 | -0.435 | 1 | -0.273* | 0.183 | -0.069 | 0.239 | -0.165 |
| C3 | 0.457 | 0.245 | 0.2 | 0.66 | 0.673 | -0.391 | -0.273 | 1 | -0.225 | 0.321* | 0.631** | 0.61** |
| G3 | 0.028 | -0.014 | -0.204 | 0.394 | 0.521 | -0.283 | 0.183 | -0.225 | 1 | 0.315* | -0.234 | -0.373** |
| ENC | 0.216 | 0.121 | 0.042 | 0.38 | 0.419 | -0.124 | -0.069 | 0.321 | 0.315 | 1 | 0.054 | -0.063 |
| CAI | 0.408 | 0.362 | 0.102 | 0.492 | 0.385 | -0.54 | 0.239 | 0.631 | -0.234 | 0.054 | 1 | 0.74** |
| CBI | 0.509 | 0.431 | 0.326 | 0.358 | 0.328 | -0.371 | -0.165 | 0.61 | -0.373 | -0.063 | 0.74 | 1 |

*A. coreanum*

|  | GC_ALL_ | GC_1_ | GC_2_ | GC_3_ | GC_3s_ | A_3_ | T_3_ | C_3_ | G_3_ | ENC | CAI | CBI |
| --- | --- | --- | --- | --- | --- | --- | --- | --- | --- | --- | --- | --- |
| GCALL | 1 | 0.776^**^ | 0.721^**^ | 0.515^**^ | 0.535^**^ | -0.355^**^ | -0.346^*^ | 0.455^**^ | 0.043 | 0.306^*^ | 0.396^**^ | 0.527^**^ |
| GC1 | 0.776^**^ | 1 | 0.403** | 0.125 | 0.291* | -0.184 | -0.241 | 0.193 | 0.031 | 0.191 | 0.298* | 0.357** |
| GC2 | 0.721^**^ | 0.403** | 1 | 0.001 | 0.08 | 0.008 | -0.403^**^ | 0.157 | -0.211 | 0.114 | 0.029 | 0.291* |
| GC3 | 0.515^**^ | 0.125 | 0.001 | 1 | 0.765** | -0.588** | -0.033 | 0.612** | 0.301* | 0.332* | 0.508** | 0.432** |
| GC3s | 0.535^**^ | 0.291* | 0.08 | 0.765** | 1 | -0.605** | -0.178 | 0.6880** | 0.559** | 0.511** | 0.368** | 0.373** |
| A3 | -0.355^**^ | -0.184 | 0.008 | -0.588** | -0.605** | 1 | -0.482** | -0.363** | -0.285* | -0.115 | -0.543** | -0.379** |
| T3 | -0.346^*^ | -0.241 | -0.403** | -0.033 | -0.178 | -0.482** | 1 | -0.262 | 0.131 | -0.196 | 0.289* | -0.14 |
| C3 | 0.455^**^ | 0.193 | 0.157 | 0.612** | 0.6880** | -0.363** | -0.262 | 1 | -0.17 | 0.393** | 0.566** | 0.617** |
| G3 | 0.043 | 0.031 | -0.211 | 0.301* | 0.559** | -0.285* | 0.131 | -0.17 | 1 | 0.352** | -0.164 | -0.295* |
| ENC | 0.306^*^ | 0.191 | 0.114 | 0.332* | 0.511** | -0.115 | -0.196 | 0.393** | 0.352** | 1 | 0.071 | 0.002 |
| CAI | 0.396^**^ | 0.298* | 0.029 | 0.508** | 0.368** | -0.543** | 0.289* | 0.566** | -0.164 | 0.071 | 1 | 0.727** |
| CBI | 0.527^**^ | 0.357** | 0.291* | 0.432** | 0.373** | -0.379** | -0.14 | 0.617** | -0.295* | 0.002 | 0.727** | 1 |

1. *delavayi*

|  | GC_ALL_ | GC_1_ | GC_2_ | GC_3_ | GC_3s_ | A_3_ | T_3_ | C_3_ | G_3_ | ENC | CAI | CBI |
| --- | --- | --- | --- | --- | --- | --- | --- | --- | --- | --- | --- | --- |
| GCALL | 1 | 0.815** | 0.778** | 0.555** | 0.532** | -0.363** | -0.345* | 0.445** | 0.025 | 0.212 | 0.399** | 0.481** |
| GC1 | 0.815** | 1 | 0.431** | 0.252 | 0.314 | -0.242 | -0.214 | 0.249 | -0.021 | 0.125 | 0.358** | 0.427** |
| GC2 | 0.778** | 0.431** | 1 | 0.158 | 0.130 | -0.035 | -0.418** | 0.193 | -0.2 | 0.035 | 0.092 | 0.298* |
| GC3 | 0.555** | 0.252 | 0.158 | 1 | 0.883** | -0.64** | -0.075 | 0.636** | 0.393** | 0.376** | 0.498** | 0.325* |
| GC3s | 0.532** | 0.314 | 0.13 | 0.883** | 1 | -0.633** | -0.161 | 0.656** | 0.518** | 0.413** | 0.374** | 0.302* |
| A3 | -0.363** | -0.242 | -0.035 | -0.64** | -0.633** | 1 | -0.447** | -0.374** | -0.275* | -0.123 | -0.542** | -0.345* |
| T3 | -0.345* | -0.214 | -0.418** | -0.075 | -0.161 | -0.447** | 1 | -0.273* | 0.185 | -0.064 | 0.251 | -0.163 |
| C3 | 0.445** | 0.249 | 0.193 | 0.636** | 0.656** | -0.374** | -0.273* | 1 | -0.252 | 0.327* | 0.629** | 0.617** |
| G3 | 0.025 | -0.021 | -0.2 | 0.393** | 0.518** | -0.275* | 0.185 | -0.252 | 1 | 0.288* | -0.248 | -0.407** |
| ENC | 0.212 | 0.125 | 0.035 | 0.376** | 0.413** | -0.123 | -0.064 | 0.327* | 0.288* | 1 | 0.059 | -0.042 |
| CAI | 0.399** | 0.358** | 0.092 | 0.498** | 0.374** | -0.542** | 0.251 | 0.629** | -0.248 | 0.059 | 1 | 0.738** |
| CBI | 0.481** | 0.427** | 0.298* | 0.325* | 0.302* | -0.345* | -0.163 | 0.617** | -0.407** | -0.042 | 0.738** | 1 |

1. *duclouxii*

|  | GC_ALL_ | GC_1_ | GC_2_ | GC_3_ | GC_3s_ | A_3_ | T_3_ | C_3_ | G_3_ | ENC | CAI | CBI |
| --- | --- | --- | --- | --- | --- | --- | --- | --- | --- | --- | --- | --- |
| GCALL | 1 | 0.804** | 0.775** | 0.539** | 0.521** | -0.339* | -0.34* | 0.437** | 0.036 | 0.235 | 0.381** | 0.459** |
| GC1 | 0.804** | 1 | 0.413** | 0.226 | 0.309* | -0.216 | -0.222 | 0.238 | -0.01 | 0.119 | 0.352** | 0.399** |
| GC2 | 0.775** | 0.413** | 1 | 0.138 | 0.143 | -0.019 | -0.412** | 0.203 | -0.179 | 0.007 | 0.068 | 0.267 |
| GC3 | 0.539** | 0.226 | 0.138 | 1 | 0.820*8 | -0.618** | -0.041 | 0.599** | 0.380** | 0.485** | 0.470** | 0.322* |
| GC3s | 0.521** | 0.309* | 0.143 | 0.820** | 1 | -0.616** | -0.168 | 0.648** | 0.549** | 0.483** | 0.346* | 0.297* |
| A3 | -0.339* | -0.216 | -0.019 | -0.618** | -0.616** | 1 | -0.472** | -0.348* | -0.303* | -0.162 | -0.53** | -0.316* |
| T3 | -0.34* | -0.222 | -0.412** | -0.041 | -0.168 | -0.472** | 1 | -0.267 | 0.163 | -0.097 | 0.279* | -0.164 |
| C3 | 0.437** | 0.238 | 0.203 | 0.599** | 0.648** | -0.348* | -0.267 | 1 | -0.227 | 0.372** | 0.59** | 0.595** |
| G3 | 0.036 | -0.01 | -0.179 | 0.380** | 0.549** | -0.303* | 0.163 | -0.227 | 1 | 0.328* | -0.224 | -0.373*8 |
| ENC | 0.235 | 0.119 | 0.007 | 0.485** | 0.483** | -0.162 | -0.097 | 0.372** | 0.328* | 1 | 0.073 | -0.027 |
| CAI | 0.381** | 0.352** | 0.068 | 0.470** | 0.346* | -0.53** | 0.279* | 0.59** | -0.224 | 0.073 | 1 | 0.717** |
| CBI | 0.459** | 0.399** | 0.267 | 0.322* | 0.297* | -0.316* | -0.164 | 0.595** | -2.984 | -0.027 | 0.717** | 1 |

1. *episcopale*

|  | GC_ALL_ | GC_1_ | GC_2_ | GC_3_ | GC_3s_ | A_3_ | T_3_ | C_3_ | G_3_ | ENC | CAI | CBI |
| --- | --- | --- | --- | --- | --- | --- | --- | --- | --- | --- | --- | --- |
| GCALL | 1 | 0.809** | 0.775** | 0.542** | 0.463** | -0.313* | -0.273* | 0.372* | 0.064 | 0.304* | 0.302* | 0.362** |
| GC1 | 0.809** | 1 | 0.419** | 0.242 | 0.207 | -0.171 | -0.22 | 0.124 | -0.004 | 0.154 | 0.26 | 0.298* |
| GC2 | 0.775** | 0.419** | 1 | 0.133 | 0.202 | -0.106 | -0.307* | 0.23 | -0.117 | 0.137 | 0.09 | 0.254 |
| GC3 | 0.542** | 0.242 | 0.133 | 1 | 0.725** | -0.491** | -0.008 | 0.543** | 0.362** | 0.445** | 0.352** | 0.220. |
| GC3s | 0.463** | 0.207 | 0.202 | 0.725** | 1 | -0.615** | -0.166 | 0.644** | 0.543** | 0.471** | 0.340* | 0.259 |
| A3 | -0.313* | -0.171 | -0.106 | -0.491** | -0.615** | 1 | -0.474** | -0.34* | -0.295* | -0.148 | -0.522** | -0.303* |
| T3 | -0.273* | -0.22 | -0.307* | -0.008 | -0.166 | -0.474** | 1 | -0.273* | 0.159 | -0.116 | 0.28* | -0.139 |
| C3 | 0.372* | 0.124 | 0.23 | 0.543** | 0.644** | -0.34* | -0.273* | 1 | -0.24 | 0.375** | 0.607** | 0.605** |
| G3 | 0.064 | -0.004 | -0.117 | 0.362** | 0.543** | -0.295* | 0.159 | -0.24 | 1 | 0.296* | -0.253- | 0.426** |
| ENC | 0.304* | 0.154 | 0.137 | 0.445** | 0.471** | -0.148 | -0.116 | 0.375** | 0.296* | 1 | 0.071 | -0.007 |
| CAI | 0.302* | 0.26 | 0.09 | 0.352** | 0.340* | -0.522** | 0.28* | 0.607** | -0.253- | 0.071 | 1 | 0.724** |
| CBI | 0.362** | 0.298* | 0.254 | 0.220. | 0.259 | -0.303* | -0.139 | 0.605** | 0.426** | -0.007 | 0.724** | 1 |

1. *finetianum*

|  | GC_ALL_ | GC_1_ | GC_2_ | GC_3_ | GC_3s_ | A_3_ | T_3_ | C_3_ | G_3_ | ENC | CAI | CBI |
| --- | --- | --- | --- | --- | --- | --- | --- | --- | --- | --- | --- | --- |
| GCALL | 1 | 0.803** | 0.751** | 0.538** | 0.523** | -0.338* | -0.323* | 0.494** | -0.024 | 0.178 | 0.410** | 0.565** |
| GC1 | 0.803** | 1 | 0.362** | 0.245 | 0.341* | -0.22 | -0.215 | 0.298* | -0.026 | 0.097 | 0.365** | 0.435** |
| GC2 | 0.751** | 0.362** | 1 | 0.122 | 0.083 | -0.055 | -0.320* | 0.202 | -0.256 | -0.048 | 0.089 | 0.360** |
| GC3 | 0.538** | 0.245 | 0.122 | 1 | 0.866** | -0.558** | -0.127 | 0.670** | 0.354** | 0.440** | 0.493** | 0.429** |
| GC3s | 0.523** | 0.341* | 0.083 | 0.866** | 1 | -0.576** | -0.213 | 0.648** | 0.529** | 0.511** | 0.358** | 0.407** |
| A3 | -0.338* | -0.22 | -0.055 | -0.558** | -0.576** | 1 | -0.473** | -0.32* | -0.251 | -0.167 | -0.504** | -0.39** |
| T3 | -0.323* | -0.215 | -0.320* | -0.127 | -0.213 | -0.473** | 1 | -0.291* | 0.122 | -0.113 | 0.266 | -0.147 |
| C3 | 0.494** | 0.298* | 0.202 | 0.670** | 0.648** | -0.32* | -0.291* | 1 | -0.247 | 0.349* | 0.616** | 0.682** |
| G3 | -0.024 | -0.026 | -0.256 | 0.354** | 0.529** | -0.251 | 0.122 | -0.247 | 1 | 0.391** | -0.233 | -0.331* |
| ENC | 0.178 | 0.097 | -0.048 | 0.440** | 0.511** | -0.167 | -0.113 | 0.349* | 0.391** | 1 | 0.032 | -0.04 |
| CAI | 0.410** | 0.365** | 0.089 | 0.493** | 0.358** | -0.504** | 0.266 | 0.616** | -0.233 | 0.032 | 1 | 0.753** |
| CBI | 0.565** | 0.435** | 0.360** | 0.429** | 0.407** | -0.39** | -0.147 | 0.682** | -0.331* | -0.04 | 0.753** | 1 |

*A. flavum*

|  | GC_ALL_ | GC_1_ | GC_2_ | GC_3_ | GC_3s_ | A_3_ | T_3_ | C_3_ | G_3_ | ENC | CAI | CBI |
| --- | --- | --- | --- | --- | --- | --- | --- | --- | --- | --- | --- | --- |
| GCALL | 1 | 0.797** | 0.758** | 0.530** | 0.505** | -0.342* | -0.313* | 0.422** | 0.022 | 0.247 | 0.393** | 0.483** |
| GC1 | 0.797** | 1 | 0.370** | 0.223 | 0.284* | -0.206 | -0.191 | 0.224 | -0.025 | 0.129 | 0.373** | 0.402** |
| GC2 | 0.758** | 0.370** | 1 | 0.118 | 0.088 | -0.037 | -0.367** | 0.158 | -0.211 | 0.030 | 0.053 | 0.286* |
| GC3 | 0.530** | 0.223 | 0.118 | 1 | 0.883** | -0.613** | -0.06 | 0.632** | 0.430** | 0.466** | 0.481** | 0.348* |
| GC3s | 0.505** | 0.284* | 0.088 | 0.883** | 1 | -0.611** | -0.154 | 0.668** | 0.533** | 0.525** | 0.375** | 0.345* |
| A3 | -0.342* | -0.206 | -0.037 | -0.613** | -0.611** | 1 | -0.490** | -0.339* | -0.301* | -0.180 | -0.526** | -0.354** |
| T3 | -0.313* | -0.191 | -0.367** | -0.06 | -0.154 | -0.490** | 1 | -0.272* | 0.181 | -0.102 | 0.272* | -0.146 |
| C3 | 0.422** | 0.224 | 0.158 | 0.632** | 0.668** | -0.339* | -0.272* | 1 | -0.218 | 0.385** | 0.598** | 0.609** |
| G3 | 0.022 | -0.025 | -0.211 | 0.430** | 0.533** | -0.301* | 0.181 | -0.218 | 1 | 0.373** | -0.206 | -0.340* |
| ENC | 0.247 | 0.129 | 0.03 | 0.466** | 0.525** | -0.18 | -0.102 | 0.385** | 0.373** | 1 | 0.082 | -0.035 |
| CAI | 0.393** | 0.373** | 0.053 | 0.481** | 0.375** | -0.526** | 0.272* | 0.598** | -0.206 | 0.082 | 1 | 0.722** |
| CBI | 0.483** | 0.402** | 0.286* | 0.348* | 0.345* | -0.354** | -0.146 | 0.609** | -0.340* | -0.035 | 0.722** | 1 |

*A. hemsleyanum*

|  | GC_ALL_ | GC_1_ | GC_2_ | GC_3_ | GC_3s_ | A_3_ | T_3_ | C_3_ | G_3_ | ENC | CAI | CBI |
| --- | --- | --- | --- | --- | --- | --- | --- | --- | --- | --- | --- | --- |
| GCALL | 1 | 0.815** | 0.784** | 0.562** | 0.275* | -0.262 | 0.037 | 0.260 | 0.018 | -0.033 | 0.392** | 0.328* |
| GC1 | 0.815** | 1 | 0.437** | 0.257 | 0.224 | -0.255 | 0.035 | 0.316* | -0.126 | -0.108 | 0.483** | 0.410** |
| GC2 | 0.784** | 0.437** | 1 | 0.176 | -0.016 | -0.02 | -0.007 | -0.037 | -0.035 | -0.1 | 0.196 | 0.179 |
| GC3 | 0.562** | 0.257 | 0.176 | 1 | 0.493** | -0.356** | 0.065 | 0.344** | 0.292* | 0.210 | 0.141 | 0.087 |
| GC3s | 0.275* | 0.224 | -0.016 | 0.493** | 1 | -0.636** | -0.163 | 0.658** | 0.516** | 0.417** | 0.373** | 0.314* |
| A3 | -0.262 | -0.255 | -0.02 | -0.356** | -0.636** | 1 | -0.443** | -0.373** | -0.281* | -0.129 | -0.531** | -0.346* |
| T3 | 0.037 | 0.035 | -0.007 | 0.065 | -0.163 | -0.443** | 1 | -0.275* | 0.186 | -0.063 | 0.240 | -0.167 |
| C3 | 0.26 | 0.316* | -0.037 | 0.344** | 0.658** | -0.373** | -0.275* | 1 | -0.250 | 0.310* | 0.631** | 0.619** |
| G3 | 0.018 | -0.126 | -0.035 | 0.292* | 0.516** | -0.281* | 0.186 | -0.25 | 1 | 0.308* | -0.254 | -0.400** |
| ENC | -0.033 | -0.108 | -0.1 | 0.21 | 0.417** | -0.129 | -0.063 | 0.310* | 0.308* | 1 | 0.04 | -0.067 |
| CAI | 0.392** | 0.483** | 0.196 | 0.141 | 0.373** | -0.531** | 0.24 | 0.631** | -0.254 | 0.04 | 1 | 0.745** |
| CBI | 0.328* | 0.410** | 0.179 | 0.087 | 0.314* | -0.346* | -0.167 | 0.619** | -0.400** | -0.067 | 0.745** | 1 |

*A. jaluense* subsp. *jaluense*

|  | GC_ALL_ | GC_1_ | GC_2_ | GC_3_ | GC_3s_ | A_3_ | T_3_ | C_3_ | G_3_ | ENC | CAI | CBI |
| --- | --- | --- | --- | --- | --- | --- | --- | --- | --- | --- | --- | --- |
| GCALL | 1 | 0.807** | 0.772** | 0.517** | 0.497** | -0.337* | -0.332* | 0.442** | 0 | 0.196 | 0.374** | 0.480** |
| GC1 | 0.807** | 1 | 0.417** | 0.214 | 0.277* | -0.210 | -0.211 | 0.224 | -0.036 | 0.048 | 0.340* | 0.399** |
| GC2 | 0.772** | 0.417** | 1 | 0.104 | 0.077 | -0.018 | -0.388** | 0.184 | -0.261 | -0.004 | 0.077 | 0.318* |
| GC3 | 0.517** | 0.214 | 0.104 | 1 | 0.890** | -0.622** | -0.059 | 0.654** | 0.437** | 0.491** | 0.450** | 0.309* |
| GC3s | 0.497** | 0.277* | 0.077 | 0.890** | 1 | -0.628** | -0.140 | 0.674** | 0.550** | 0.528** | 0.324* | 0.286* |
| A3 | -0.337* | -0.21 | -0.018 | -0.622** | -0.628** | 1 | -0.482** | -0.365** | -0.310* | -0.192 | -0.502** | -0.328* |
| T3 | -0.332* | -0.211 | -0.388** | -0.059 | -0.14 | -0.482** | 1 | -0.246 | 0.171 | -0.072 | 0.299* | -0.121 |
| C3 | 0.442** | 0.224 | 0.184 | 0.654** | 0.674** | -0.365** | -0.246 | 1 | -0.190 | 0.389** | 0.569** | 0.584** |
| G3 | 0 | -0.036 | -0.261 | 0.437** | 0.550** | -0.310* | 0.171 | -0.19 | 1 | 0.393** | -0.229 | -0.378** |
| ENC | 0.196 | 0.048 | -0.004 | 0.491** | 0.528** | -0.192 | -0.072 | 0.389** | 0.393** | 1 | 0.031 | -0.059 |
| CAI | 0.374** | 0.340* | 0.077 | 0.450** | 0.324* | -0.502** | 0.299* | 0.569** | -0.229 | 0.031 | 1 | 0.730** |
| CBI | 0.480** | 0.399** | 0.318* | 0.309* | 0.286* | -0.328* | -0.121 | 0.584** | -0.378** | -0.059 | 0.730** | 1 |

*A. japonicum* subsp. *napiforme*

|  | GC_ALL_ | GC_1_ | GC_2_ | GC_3_ | GC_3s_ | A_3_ | T_3_ | C_3_ | G_3_ | ENC | CAI | CBI |
| --- | --- | --- | --- | --- | --- | --- | --- | --- | --- | --- | --- | --- |
| GCALL | 1 | 0.807** | 0.772** | 0.522** | 0.536** | -0.380** | -0.446** | 0.474** | -0.043 | 0.251 | 0.348* | 0.525** |
| GC1 | 0.807** | 1 | 0.416** | 0.217 | 0.266 | -0.14 | -0.388** | 0.396** | -0.226 | 0.213 | 0.408** | 0.538** |
| GC2 | 0.772** | 0.416** | 1 | 0.112 | 0.127 | -0.140 | -0.429** | 0.077 | -0.185 | -0.052 | 0.005 | 0.262 |
| GC3 | 0.522** | 0.217 | 0.112 | 1 | 0.946** | -0.668** | -0.066 | 0.653** | 0.482** | 0.478** | 0.385** | 0.318* |
| GC3s | 0.536** | 0.266 | 0.127 | 0.946** | 1 | -0.626** | -0.139 | 0.662** | 0.547** | 0.512** | 0.330* | 0.291* |
| A3 | -0.380** | -0.14 | -0.14 | -0.668** | -0.626** | 1 | -0.485** | -0.357** | -0.308* | -0.177 | -0.503** | -0.323* |
| T3 | -0.446** | -0.388** | -0.429** | -0.066 | -0.139 | -0.485** | 1 | -0.244 | 0.171 | -0.087 | 0.295* | -0.131 |
| C3 | 0.474** | 0.396** | 0.077 | 0.653** | 0.662** | -0.357** | -0.244 | 1 | -0.209 | 0.377** | 0.575** | 0.584** |
| G3 | -0.043 | -0.226 | -0.185 | 0.482** | 0.547** | -0.308* | 0.171 | -0.209 | 1 | 0.373* | -0.228 | -0.372** |
| ENC | 0.251 | 0.213 | -0.052 | 0.478** | 0.512** | -0.177 | -0.087 | 0.377** | 0.373* | 1 | 0.028 | -0.067 |
| CAI | 0.348* | 0.408** | 0.005 | 0.385** | 0.330* | -0.503** | 0.295* | 0.575** | -0.228 | 0.028 | 1 | 0.725** |
| CBI | 0.525** | 0.538** | 0.262 | 0.318* | 0.291* | -0.323* | -0.131 | 0.584** | -0.372** | -0.067 | 0.725** | 1 |

*A. kusnezoffii*

|  | GC_ALL_ | GC_1_ | GC_2_ | GC_3_ | GC_3s_ | A_3_ | T_3_ | C_3_ | G_3_ | ENC | CAI | CBI |
| --- | --- | --- | --- | --- | --- | --- | --- | --- | --- | --- | --- | --- |
| GCALL | 1 | 0.805** | 0.774** | 0.526** | 0.5** | -0.339* | -0.338* | 0.445** | 0.003 | 0.219 | 0.376** | 0.482** |
| GC1 | 0.805** | 1 | 0.417** | 0.214 | 0.283* | -0.212 | -0.220 | 0.230 | -0.034 | 0.066 | 0.339* | 0.398** |
| GC2 | 0.774** | 0.417** | 1 | 0.119 | 0.083 | -0.019 | -0.393** | 0.189 | -0.25 | 0.026 | 0.076 | 0.318* |
| GC3 | 0.526** | 0.214 | 0.119 | 1 | 0.880** | -0.623** | -0.061 | 0.648** | 0.422** | 0.486** | 0.4598* | 0.320* |
| GC3s | 0.5** | 0.283* | 0.083 | 0.880** | 1 | -0.637** | -0.141 | 0.661** | 0.552** | 0.516** | 0.329* | 0.298* |
| A3 | -0.339* | -0.212 | -0.019 | -0.623** | -0.637** | 1 | -0.469** | -0.357** | -0.325* | -0.179 | -0.495** | -0.325* |
| T3 | -0.338* | -0.22 | -0.393** | -0.061 | -0.141 | -0.469** | 1 | -0.252 | 0.178 | -0.084 | 0.286* | -0.146 |
| C3 | 0.445** | 0.23 | 0.189 | 0.648** | 0.661** | -0.357** | -0.252 | 1 | -0.206 | 0.379** | 0.576** | 0.591** |
| G3 | 0.003 | -0.034 | -0.25 | 0.422** | 0.552** | -0.325* | 0.178 | -0.206 | 1 | 0.382** | -0.227 | 0.370** |
| ENC | 0.219 | 0.066 | 0.026 | 0.486** | 0.516** | -0.179 | -0.084 | 0.379** | 0.382** | 1 | 0.024 | -0.079 |
| CAI | 0.376** | 0.339* | 0.076 | 0.4598* | 0.329* | -0.495** | 0.286* | 0.576** | -0.227 | 0.024 | 1 | 0.724** |
| CBI | 0.482** | 0.398** | 0.318* | 0.320* | 0.298* | -0.325* | -0.146 | 0.591** | 0.370** | -0.079 | 0.724** | 1 |

*A. longecassidatum*

|  | GC_ALL_ | GC_1_ | GC_2_ | GC_3_ | GC_3s_ | A_3_ | T_3_ | C_3_ | G_3_ | ENC | CAI | CBI |
| --- | --- | --- | --- | --- | --- | --- | --- | --- | --- | --- | --- | --- |
| GCALL | 1 | 0.795** | 0.750** | 0.483** | 0.469** | -0.275* | --0.237 | 0.399** | 0.063 | 0.239 | 0.361** | 0.456** |
| GC1 | 0.795** | 1 | 0.358** | 0.181 | 0.315* | -0.229 | -0.135 | 0.265 | 0.02 | 0.097 | 0.399** | 0.426** |
| GC2 | 0.750** | 0.358** | 1 | 0.08 | 0.102 | -0.022 | -0.222 | 0.079 | -0.074 | 0.08 | 0.124 | 0.245 |
| GC3 | 0.483** | 0.181 | 0.08 | 1 | 0.673** | -0.430** | -0.102 | 0.587** | 0.235 | 0.342* | 0.244 | 0.259 |
| GC3s | 0.469** | 0.315* | 0.102 | 0.673** | 1 | -0.551** | -0.206 | 0.653** | 0.562** | 0.552** | 0.325* | 0.340* |
| A3 | -0.275* | -0.229 | -0.022 | -0.430** | -0.551** | 1 | -0.526** | -0.295* | -0.283* | -0.142 | -0.494** | -0.346* |
| T3 | 0.237 | -0.135 | -0.222 | -0.102 | -0.206 | -0.526** | 1 | -0.273* | 0.099 | -0.174 | 0.291* | -0.106 |
| C3 | 0.399** | 0.265 | 0.079 | 0.587** | 0.653** | -0.295* | -0.273* | 1 | -0.206 | 0.385** | 0.594** | 0.631** |
| G3 | 0.063 | 0.02 | -0.074 | 0.235 | 0.562** | -0.283* | 0.099 | -0.206 | 1 | 0.402** | -0.234 | -0.337* |
| ENC | 0.239 | 0.097 | 0.08 | 0.342* | 0.552** | -0.142 | -0.174 | 0.385** | 0.402** | 1 | 0.02 | -0.032 |
| CAI | 0.361** | 0.399** | 0.124 | 0.244 | 0.325* | -0.494** | 0.291* | 0.594** | -0.234 | 0.02 | 1 | 0.746** |
| CBI | 0.456** | 0.426** | 0.245 | 0.259 | 0.340* | -0.346* | -0.106 | 0.631** | -0.337* | -0.032 | 0.746** | 1 |

1. *monanthum*

|  | GC_ALL_ | GC_1_ | GC_2_ | GC_3_ | GC_3s_ | A_3_ | T_3_ | C_3_ | G_3_ | ENC | CAI | CBI |
| --- | --- | --- | --- | --- | --- | --- | --- | --- | --- | --- | --- | --- |
| GCALL | 1 | 0.807** | 0.772** | 0.523** | 0.516** | -0.355** | -0.328* | 0.442** | 0.024 | 0.255 | 0.378** | 0.477** |
| GC1 | 0.807** | 1 | 0.414** | 0.226 | 0.305* | -0.224 | -0.215 | 0.232 | -0.008 | 0.099 | 0.343* | 0.399** |
| GC2 | 0.772** | 0.414** | 1 | 0.108 | 0.091 | -0.035 | -0.380** | 0.188 | -0.239 | 0.072 | 0.076 | 0.311* |
| GC3 | 0.523** | 0.226 | 0.108 | 1 | 0.890** | -0.631** | -0.056 | 0.640** | 0.436** | 0.474** | 0.462** | 0.313* |
| GC3s | 0.516** | 0.305* | 0.091 | 0.890** | 1 | -0.630** | -0.126 | 0.654** | 0.558** | 0.515** | 0.354** | 0.297* |
| A3 | -0.355** | -0.224 | -0.035 | -0.631** | -0.630** | 1 | -0.491** | -0.349* | -0.323* | -0.192 | -0.510** | -0.320* |
| T3 | -0.328* | -0.215 | -0.380** | -0.056 | -0.126 | -0.491** | 1 | -0.245 | 0.183 | -0.081 | 0.282* | -0.136 |
| C3 | 0.442** | 0.232 | 0.188 | 0.640** | 0.654** | -0.349* | -0.245 | 1 | -0.208 | 0.354* | 0.597** | 0.600** |
| G3 | 0.024 | -0.008 | -0.239 | 0.436** | 0.558** | -0.323* | 0.183 | -0.208 | 1 | 0.394** | -0.209 | -0.366** |
| ENC | 0.255 | 0.099 | 0.072 | 0.474** | 0.515** | -0.192 | -0.081 | 0.354* | 0.394** | 1 | 0.030 | -0.075 |
| CAI | 0.378** | 0.343* | 0.076 | 0.462** | 0.354** | -0.510** | 0.282* | 0.597** | -0.209 | 0.03 | 1 | 0.729** |
| CBI | 0.477** | 0.399** | 0.311* | 0.313* | 0.297* | -0.320* | -0.136 | 0.600** | -0.366** | -0.075 | 0.729** | 1 |

*A. nagarum*

|  | GC_ALL_ | GC_1_ | GC_2_ | GC_3_ | GC_3s_ | A_3_ | T_3_ | C_3_ | G_3_ | ENC | CAI | CBI |
| --- | --- | --- | --- | --- | --- | --- | --- | --- | --- | --- | --- | --- |
| GCALL | 1 | 0.807** | 0.772** | 0.523** | 0.516** | -0.355** | -0.328* | 0.442* | 0.024 | 0.255 | 0.378** | 0.477** |
| GC1 | 0.807** | 1 | 0.414** | 0.226 | 0.305* | -0.224 | -0.215 | 0.232 | -0.008 | 0.099 | 0.343* | 0.399** |
| GC2 | 0.772** | 0.414** | 1 | 0.108 | 0.091 | -0.035 | -0.38** | 0.188 | -0.239 | 0.072 | 0.076 | 0.311* |
| GC3 | 0.523** | 0.226 | 0.108 | 1 | 0.890** | -0.631** | -0.056 | 0.640** | 0.436** | 0.474** | 0.462** | 0.313* |
| GC3s | 0.516** | 0.305* | 0.091 | 0.890** | 1 | -0.630** | -0.126 | 0.654** | 0.558** | 0.515** | 0.354** | 0.297* |
| A3 | -0.355** | -0.224 | -0.035 | -0.631** | -0.630** | 1 | -0.491** | -0.349* | -0.323* | -0.192- | 0.510** | -0.320* |
| T3 | -0.328* | -0.215 | -0.38** | -0.056 | -0.126 | -0.491** | 1 | -0.245 | 0.183 | -0.081 | 0.282* | -0.136 |
| C3 | 0.442* | 0.232 | 0.188 | 0.640** | 0.654** | -0.349* | -0.245 | 1 | -0.208 | 0.354** | 0.597** | 0.600** |
| G3 | 0.024 | -0.008 | -0.239 | 0.436** | 0.558** | -0.323* | 0.183 | -0.208 | 1 | 0.394** | -0.209 | -0.366** |
| ENC | 0.255 | 0.099 | 0.072 | 0.474** | 0.515** | -0.192- | -0.081 | 0.354** | 0.394** | 1 | 0.03 | -0.075 |
| CAI | 0.378** | 0.343* | 0.076 | 0.462** | 0.354** | 0.510** | 0.282* | 0.597** | -0.209 | 0.03 | 1 | 0.729** |
| CBI | 0.477** | 0.399** | 0.311* | 0.313* | 0.297* | -0.320* | -0.136 | 0.600** | -0.366** | -0.075 | 0.729** | 1 |

1. *ouvrardianum*

|  | GC_ALL_ | GC_1_ | GC_2_ | GC_3_ | GC_3s_ | A_3_ | T_3_ | C_3_ | G_3_ | ENC | CAI | CBI |
| --- | --- | --- | --- | --- | --- | --- | --- | --- | --- | --- | --- | --- |
| GCALL | 1 | 0.808** | 0.774** | 0.536** | 0.514** | -0.354** | -0.317* | 0.423** | 0.038 | 0.250 | 0.381** | 0.447** |
| GC1 | 0.807** | 1 | 0.416** | 0.237 | 0.292* | -0.234 | -0.190 | 0.231 | -0.029 | 0.141 | 0.359** | 0.411** |
| GC2 | 0.772** | 0.414** | 1 | 0.128 | 0.107 | -0.031 | -0.387** | 0.164 | -0.190 | 0.057 | 0.067 | 0.265 |
| GC3 | 0.523** | 0.226 | 0.108 | 1 | 0.888** | -0.624** | -0.058 | 0.629** | 0.432** | 0.424** | 0.466** | 0.287* |
| GC3s | 0.516** | 0.305* | 0.091 | 0.890** | 1 | -0.622** | -0.148 | 0.647** | 0.543** | 0.472** | 0.353** | 0.275* |
| A3 | -0.355** | -0.224 | -0.035 | -0.631** | -0.630** | 1 | -0.481** | -0.345* | -0.301* | -0.158 | -0.527** | -0.309* |
| T3 | -0.328* | -0.215 | -0.38** | -0.056 | -0.126 | -0.491** | 1 | -0.265 | 0.175 | -0.1 | 0.283* | -0.142 |
| C3 | 0.442* | 0.232 | 0.188 | 0.640** | 0.654** | -0.349* | -0.245 | 1 | -0.235 | 0.375** | 0.607** | 0.606** |
| G3 | 0.024 | -0.008 | -0.239 | 0.436** | 0.558** | -0.323* | 0.183 | -0.208 | 1 | 0.299* | -0.235 | -0.408** |
| ENC | 0.255 | 0.099 | 0.072 | 0.474** | 0.515** | -0.192- | -0.081 | 0.354** | 0.394** | 1 | 0.088 | 0.002 |
| CAI | 0.378** | 0.343* | 0.076 | 0.462** | 0.354** | 0.510** | 0.282* | 0.597** | -0.209 | 0.03 | 1 | 0.724** |
| CBI | 0.477** | 0.399** | 0.311* | 0.313* | 0.297* | -0.320* | -0.136 | 0.600** | -0.366** | -0.075 | 0.729** | 1 |

*A. pendulum*

|  | GC_ALL_ | GC_1_ | GC_2_ | GC_3_ | GC_3s_ | A_3_ | T_3_ | C_3_ | G_3_ | ENC | CAI | CBI |
| --- | --- | --- | --- | --- | --- | --- | --- | --- | --- | --- | --- | --- |
| GCALL | 1 | 0.799** | 0.760** | 0.528** | 0.503** | -0.339* | -0.316* | 0.423** | 0.02 | 0.248 | 0.390** | 0.482** |
| GC1 | 0.799** | 1 | 0.377** | 0.219 | 0.280* | -0.199 | -0.196 | 0.224 | -0.028 | 0.127 | 0.370** | 0.401** |
| GC2 | 0.760** | 0.377** | 1 | 0.120 | 0.09 | -0.037 | -0.370** | 0.160 | -0.221 | 0.038 | 0.052 | 0.287* |
| GC3 | 0.528** | 0.219 | 0.12 | 1 | 0.883** | -0.614** | -0.059 | 0.633** | 0.428** | 0.461** | 0.481** | 0.346* |
| GC3s | 0.503** | 0.280* | 0.09 | 0.883** | 1 | -0.612** | -0.154 | 0.669** | 0.531** | 0.520** | 0.375** | 0.345* |
| A3 | -0.339* | -0.199 | -0.037 | -0.614** | -0.612** | 1 | -0.487** | -0.340* | -0.3* | -0.178 | -0.526** | -0.354** |
| T3 | -0.316* | -0.196 | -0.370** | -0.059 | -0.154 | -0.487** | 1 | -0.271* | 0.182 | -0.109 | 0.274* | -0.148 |
| C3 | 0.423** | 0.224 | 0.16 | 0.633** | 0.669** | -0.340* | -0.271* | 1 | -0.221 | 0.382** | 0.598** | 0.610** |
| G3 | 0.02 | -0.028 | -0.221 | 0.428** | 0.531** | -0.3* | 0.182 | -0.221 | 1 | 0.363** | -0.207 | -0.342* |
| ENC | 0.248 | 0.127 | 0.038 | 0.461** | 0.520** | -0.178 | -0.109 | 0.382** | 0.363** | 1 | 0.078 | -0.036 |
| CAI | 0.390** | 0.370** | 0.052 | 0.481** | 0.375** | -0.526** | 0.274* | 0.598** | -0.207 | 0.078 | 1 | 0.721** |
| CBI | 0.482** | 0.401** | 0.287* | 0.346* | 0.345* | -0.354** | -0.148 | 0.610** | -0.342* | -0.036 | 0.721** | 1 |

*A. piepunense*

|  | GC_ALL_ | GC_1_ | GC_2_ | GC_3_ | GC_3s_ | A_3_ | T_3_ | C_3_ | G_3_ | ENC | CAI | CBI |
| --- | --- | --- | --- | --- | --- | --- | --- | --- | --- | --- | --- | --- |
| GCALL | 1 | 0.811** | 0.772** | 0.536** | 0.519** | -0.354** | -0.322 | 0.419** | 0.04 | 0.234 | 0.377** | 0.438** |
| GC1 | 0.811** | 1 | 0.417** | 0.243 | 0.291* | -0.232 | -0.195 | 0.228 | -0.03 | 0.129 | 0.355** | 0.406** |
| GC2 | 0.772** | 0.417** | 1 | 0.1213 | 0.110 | -0.029 | -0.392** | 0.164 | -0.186 | 0.044 | 0.067 | 0.258 |
| GC3 | 0.536** | 0.243 | 0.1213 | 1 | 0.899** | -0.634** | -0.057 | 0.625** | 0.435** | 0.418** | 0.461** | 0.281* |
| GC3s | 0.519** | 0.291* | 0.11 | 0.899** | 1 | -0.622** | -0.147 | 0.644** | 0.535** | 0.468** | 0.351* | 0.263 |
| A3 | -0.354** | -0.232 | -0.029 | -0.634** | -0.622** | 1 | -0.480** | -0.352** | -0.292* | -0.161 | -0.531** | -0.303* |
| T3 | -0.322 | -0.195 | -0.392** | -0.057 | -0.147 | -0.480** | 1 | -0.256 | -0.173 | -0.085 | 0.285* | -0.138 |
| C3 | 0.419** | 0.228 | 0.164 | 0.625** | 0.644** | -0.352** | -0.256 | 1 | -0.25 | 0.383** | 0.612** | 0.607** |
| G3 | 0.04 | -0.03 | -0.186 | 0.435** | 0.535** | -0.292* | -0.173 | -0.25 | 1 | 0.285* | -0.248 | -0.426** |
| ENC | 0.234 | 0.129 | 0.044 | 0.418** | 0.468** | -0.161 | -0.085 | 0.383** | 0.285* | 1 | 0.097 | 0.008 |
| CAI | 0.377** | 0.355** | 0.067 | 0.461** | 0.351* | -0.531** | 0.285* | 0.612** | -0.248 | 0.097 | 1 | 0.723** |
| CBI | 0.438** | 0.406** | 0.258 | 0.281* | 0.263 | -0.303* | -0.138 | 0.607** | -0.426** | 0.008 | 0.723** | 1 |

1. *pseudolaeve*

|  | GC_ALL_ | GC_1_ | GC_2_ | GC_3_ | GC_3s_ | A_3_ | T_3_ | C_3_ | G_3_ | ENC | CAI | CBI |
| --- | --- | --- | --- | --- | --- | --- | --- | --- | --- | --- | --- | --- |
| GCALL | 1 | 0.799** | 0.754** | 0.491** | 0.543** | -0.366** | -0.441** | 0.478** | -0.028 | 0.315* | 0.348* | 0.556** |
| GC1 | 0.799** | 1 | 0.363** | 0.203 | 0.285* | -0.134 | -0.362** | 0.435** | -0.222 | 0.241 | 0.439** | 0.566** |
| GC2 | 0.754** | 0.363** | 1 | 0.072 | 0.150 | -0.166 | -0.394** | 0.046 | -0.113 | 0.035 | -0.032 | 0.238 |
| GC3 | 0.491** | 0.203 | 0.072 | 1 | 0.901** | -0.599** | -0.106 | 0.641** | 0.442** | 0.487** | 0.380** | 0.366** |
| GC3s | 0.543** | 0.285* | 0.15 | 0.901** | 1 | -0.576** | -0.197 | 0.641** | 0.562** | 0.644** | 0.332* | 0.348* |
| A3 | -0.366** | -0.134 | -0.166 | -0.599** | -0.576** | 1 | -0.493** | -0.320* | -0.279* | -0.167 | -0.487** | -0.355** |
| T3 | -0.441** | -0.362** | -0.394** | -0.106 | -0.197 | -0.493** | 1 | -0.256 | 0.102 | -0.155 | 0.285* | -0.115 |
| C3 | 0.478** | 0.435** | 0.046 | 0.641** | 0.641** | -0.320* | -0.256 | 1 | -0.219 | 0.382** | 0.610** | 0.651** |
| G3 | -0.028 | -0.222 | -0.113 | 0.442** | 0.562** | -0.279* | 0.102 | -0.219 | 1 | 0.3868* | -0.235 | -0.343* |
| ENC | 0.315* | 0.241 | 0.035 | 0.487** | 0.644** | -0.167 | -0.155 | 0.382** | 0.3868* | 1 | 0.025 | -0.038 |
| CAI | 0.348* | 0.439** | -0.032 | 0.380** | 0.332* | -0.487** | 0.285* | 0.610** | -0.235 | 0.025 | 1 | 0.749** |
| CBI | 0.556** | 0.566** | 0.238 | 0.366** | 0.348* | -0.355** | -0.115 | 0.651** | -0.343* | -0.038 | 0.749** | 1 |

*A. puchonroenicum*

|  | GC_ALL_ | GC_1_ | GC_2_ | GC_3_ | GC_3s_ | A_3_ | T_3_ | C_3_ | G_3_ | ENC | CAI | CBI |
| --- | --- | --- | --- | --- | --- | --- | --- | --- | --- | --- | --- | --- |
| GCALL | 1 | 0.798** | 0.740** | 0.472** | 0.441** | -0.280* | -0.286* | 0.417** | -0.023 | 0.213 | 0.395** | 0.511** |
| GC1 | 0.798** | 1 | 0.352** | 0.174 | 0.249 | -0.172 | -0.161 | 0.232 | -0.058 | 0.039 | 0.372** | 0.428** |
| GC2 | 0.740** | 0.352** | 1 | 0.039 | -0.009 | 0.005 | -0.288* | 0.103 | -0.264 | 0.023 | 0.053 | 0.280* |
| GC3 | 0.472** | 0.174 | 0.039 | 1 | 0.869** | -0.531** | -0.126 | 0.661** | 0.421** | 0.505** | 0.457*8 | 0.357** |
| GC3s | 0.441** | 0.249 | -0.009 | 0.869** | 1 | -0.546** | -0.211 | 0.654** | 0.577** | 0.566** | 0.326* | 0.345* |
| A3 | -0.280* | -0.172 | 0.005 | -0.531** | -0.546** | 1 | -0.533** | -0.298* | -0.281* | -0.158 | -0.494** | -0.339* |
| T3 | -0.286* | -0.161 | -0.288* | -0.126 | -0.211 | -0.533** | 1 | -0.273* | 0.08 | -0.174 | 0.284 | -0.11 |
| C3 | 0.417** | 0.232 | 0.103 | 0.661** | 0.654** | -0.298* | -0.273* | 1 | -0.187 | 0.407** | 0.597** | 0.643** |
| G3 | -0.023 | -0.058 | -0.264 | 0.421** | 0.577** | -0.281* | 0.08 | -0.187 | 1 | 0.404** | -0.231 | -0.3288 |
| ENC | 0.213 | 0.039 | 0.023 | 0.505** | 0.566** | -0.158 | -0.174 | 0.407** | 0.404** | 1 | 0.028 | -0.019 |
| CAI | 0.395** | 0.372** | 0.053 | 0.457*8 | 0.326* | -0.494** | 0.284 | 0.597** | -0.231 | 0.028 | 1 | 0.752** |
| CBI | 0.511** | 0.428** | 0.280* | 0.357** | 0.345* | -0.339* | -0.11 | 0.643** | -0.3288 | -0.019 | 0.752** | 1 |

*A. quelpaertense*

|  | GC_ALL_ | GC_1_ | GC_2_ | GC_3_ | GC_3s_ | A_3_ | T_3_ | C_3_ | G_3_ | ENC | CAI | CBI |
| --- | --- | --- | --- | --- | --- | --- | --- | --- | --- | --- | --- | --- |
| GCALL | 1 | 0.795** | 0.754** | 0.530** | 0.498** | -0.318* | -0.328* | 0.450** | 0.001 | 0.261 | 0.383** | 0.517** |
| GC1 | 0.795** | 1 | 0.355** | 0.228 | 0.302* | -0.209 | -0.197 | 0.273* | -0.045 | 0.088 | 0.373** | 0.448** |
| GC2 | 0.754** | 0.355** | 1 | 0.119 | 0.070 | -0.032 | -0.346* | 0.152 | -0.222 | 0.09 | 0.048 | 0.294* |
| GC3 | 0.530** | 0.228 | 0.119 | 1 | 0.869** | -0.550** | -0.121 | 0.649** | 0.408** | 0.481** | 0.461** | 0.362** |
| GC3s | 0.498** | 0.302* | 0.07 | 0.869** | 1 | -0.576** | -0.196 | 0.641** | 0.563** | 0.546** | 0.334* | 0.350* |
| A3 | -0.318* | -0.209 | -0.032 | -0.550** | -0.576** | 1 | -0.494** | -0.320* | -0.279* | -0.170 | -0.490** | -0.357** |
| T3 | -0.328* | -0.197 | -0.346* | -0.121 | -0.196 | -0.494** | 1 | -0.254 | 0.101 | -0.156 | 0.286* | -0.114 |
| C3 | 0.450** | 0.273* | 0.152 | 0.649** | 0.641** | -0.320* | -0.254 | 1 | -0.217 | 0.382** | 0.610** | 0.648** |
| G3 | 0.001 | -0.045 | -0.222 | 0.408** | 0.563** | -0.279* | 0.101 | -0.217 | 1 | 0.390** | -0.232 | -0.336* |
| ENC | 0.261 | 0.088 | 0.09 | 0.481** | 0.546** | -0.17 | -0.156 | 0.382** | 0.390** | 1 | 0.028 | -0.029 |
| CAI | 0.383** | 0.373** | 0.048 | 0.461** | 0.334* | -0.490** | 0.286* | 0.610** | -0.232 | 0.028 | 1 | 0.749** |
| CBI | 0.517** | 0.448** | 0.294* | 0.362** | 0.350* | -0.357** | -0.114 | 0.648** | -0.336* | -0.029 | 0.749** | 1 |

1. *ramulosum*

|  | GC_ALL_ | GC_1_ | GC_2_ | GC_3_ | GC_3s_ | A_3_ | T_3_ | C_3_ | G_3_ | ENC | CAI | CBI |
| --- | --- | --- | --- | --- | --- | --- | --- | --- | --- | --- | --- | --- |
| GCALL | 1 | 0.809** | 0.775** | 0.537** | 0.516** | -0.353** | -0.321* | 0.422** | 0.04 | 0.220 | 0.379** | 0.437** |
| GC1 | 0.809** | 1 | 0.417** | 0.237 | 0.291* | -0.234 | -0.193 | 0.233 | -0.033 | 0.124 | 0.358** | 0.409** |
| GC2 | 0.775** | 0.417** | 1 | 0.131 | 0.111 | -0.03 | -0.389** | 0.164 | -0.182 | 0.023 | 0.066 | 0.254 |
| GC3 | 0.537** | 0.237 | 0.131 | 1 | 0.889** | -0.623** | -0.063 | 0.625** | 0.432** | 0.414** | 0.464** | 0.277* |
| GC3s | 0.516** | 0.291* | 0.111 | 0.889** | 1 | -0.620** | -0.152 | 0.642** | 0.544** | 0.459** | 0.349* | 0.261 |
| A3 | -0.353** | -0.234 | -0.03 | -0.623** | -0.620** | 1 | -0.479** | -0.344** | -0.299* | -0.156 | -0.529** | -0.303* |
| T3 | -0.321* | -0.193 | -0.389** | -0.063 | -0.152 | -0.479** | 1 | -0.266 | 0.171 | -0.079 | 0.282* | -0.139 |
| C3 | 0.422** | 0.233 | 0.164 | 0.625** | 0.642** | -0.344** | -0.266 | 1 | -0.241 | 0.379** | 0.609** | 0.607** |
| G3 | 0.04 | -0.033 | -0.182 | 0.432** | 0.544** | -0.299* | 0.171 | -0.241 | 1 | 0.284* | -0.241 | -0.423** |
| ENC | 0.22 | 0.124 | 0.023 | 0.414** | 0.459** | -0.156 | -0.079 | 0.379** | 0.284* | 1 | 0.093 | 0.014 |
| CAI | 0.379** | 0.358** | 0.066 | 0.464** | 0.349* | -0.529** | 0.282* | 0.609** | -0.241 | 0.093 | 1 | 0.723** |
| CBI | 0.437** | 0.409** | 0.254 | 0.277* | 0.261 | -0.303* | -0.139 | 0.607** | -0.423** | 0.014 | 0.723** | 1 |

*A. reclinatum*

|  | GC_ALL_ | GC_1_ | GC_2_ | GC_3_ | GC_3s_ | A_3_ | T_3_ | C_3_ | G_3_ | ENC | CAI | CBI |
| --- | --- | --- | --- | --- | --- | --- | --- | --- | --- | --- | --- | --- |
| GCALL | 1 | 0.798** | 0.739** | 0.544** | 0.541** | -0.381** | -0.274* | 0.487** | -0.004 | 0.239 | 0.457** | 0.537** |
| GC1 | 0.798** | 1 | 0.345* | 0.230 | 0.340* | -0.229 | -0.169 | 0.291* | -0.018 | 0.095 | 0.403** | 0.440** |
| GC2 | 0.739** | 0.345* | 1 | 0.129 | 0.099 | -0.101 | -0.286* | 0.186 | -0.234 | 0.034 | 0.116 | 0.297* |
| GC3 | 0.544** | 0.23 | 0.129 | 1 | 0.864** | -0.579** | -0.106 | 0.662** | 0.352* | 0.480** | 0.507** | 0.418** |
| GC3s | 0.541** | 0.340* | 0.099 | 0.864** | 1 | -0.583** | -0.217 | 0.658** | 0.514** | 0.538** | 0.358** | 0.415** |
| A3 | -0.381** | -0.229 | -0.101 | -0.579** | -0.583** | 1 | -0.463** | -0.340* | -0.230 | -0.155 | -0.488** | -0.410** |
| T3 | -0.274* | -0.169 | -0.286* | -0.106 | -0.217 | -0.463** | 1 | -0.286* | 0.112 | -0.178 | 0.236 | -0.116 |
| C3 | 0.487** | 0.291* | 0.186 | 0.662** | 0.658** | -0.340* | -0.286* | 1 | -0.251 | 0.366** | 0.631** | 0.689** |
| G3 | -0.004 | -0.018 | -0.234 | 0.352* | 0.514** | -0.23 | 0.112 | -0.251 | 1 | 0.400** | -0.263 | -0.339* |
| ENC | 0.239 | 0.095 | 0.034 | 0.480** | 0.538** | -0.155 | -0.178 | 0.366** | 0.400** | 1 | 0.004 | -0.023 |
| CAI | 0.457** | 0.403** | 0.116 | 0.507** | 0.358** | -0.488** | 0.236 | 0.631** | -0.263 | 0.004 | 1 | 0.787** |
| CBI | 0.537** | 0.440** | 0.297* | 0.418** | 0.415** | -0.410** | -0.116 | 0.689** | -0.339* | -0.023 | 0.787** | 1 |

1. *scaposum*

|  | GC_ALL_ | GC_1_ | GC_2_ | GC_3_ | GC_3s_ | A_3_ | T_3_ | C_3_ | G_3_ | ENC | CAI | CBI |
| --- | --- | --- | --- | --- | --- | --- | --- | --- | --- | --- | --- | --- |
| GCALL | 1 | 0.802** | 0.746** | 0.524** | 0.503** | -0.343* | -0.315* | 0.474** | -0.014 | 0.240 | 0.401** | 0.522** |
| GC1 | 0.802** | 1 | 0.359** | 0.227 | 0.308* | -0.223 | -0.196 | 0.282* | -0.044 | 0.085 | 0.363** | 0.428** |
| GC2 | 0.746** | 0.359** | 1 | 0.102 | 0.062 | -0.042 | -0.336* | 0.178 | -0.256 | 0.050 | 0.070 | 0.312* |
| GC3 | 0.524** | 0.227 | 0.102 | 1 | 0.872** | -0.575** | -0.102 | 0.659** | 0.408** | 0.481** | 0.486** | 0.375** |
| GC3s | 0.503** | 0.308* | 0.062 | 0.872** | 1 | -0.588** | -0.912 | 0.652** | 0.560** | 0.545** | 0.352** | 0.347* |
| A3 | -0.343* | -0.223 | -0.042 | -0.575** | -0.588** | 1 | -0.486** | -0.333* | -0.279* | -0.175 | -0.516** | -0.354** |
| T3 | -0.315* | -0.196 | -0.336* | -0.102 | -0.912 | -0.486** | 1 | -0.265 | 0.110 | -0.145 | 0.294* | -0.125 |
| C3 | 0.474** | 0.282* | 0.178 | 0.659** | 0.652** | -0.333* | -0.265 | 1 | -0.208 | 0.373** | 0.616** | 0.645** |
| G3 | -0.014 | -0.044 | -0.256 | 0.408** | 0.560** | -0.279* | 0.11 | -0.208 | 1 | 0.407** | -0.218 | -0.335* |
| ENC | 0.24 | 0.085 | 0.05 | 0.481** | 0.545** | -0.175 | -0.145 | 0.373** | 0.407** | 1 | 0.034 | -0.033 |
| CAI | 0.401** | 0.363** | 0.07 | 0.486** | 0.352** | -0.516** | 0.294* | 0.616** | -0.218 | 0.034 | 1 | 0.733** |
| CBI | 0.522** | 0.428** | 0.312* | 0.375** | 0.347* | -0.354** | -0.125 | 0.645** | -0.335* | -0.033 | 0.733** | 1 |

*A. scaposum* var. *vaginatum*

|  | GC_ALL_ | GC_1_ | GC_2_ | GC_3_ | GC_3s_ | A_3_ | T_3_ | C_3_ | G_3_ | ENC | CAI | CBI |
| --- | --- | --- | --- | --- | --- | --- | --- | --- | --- | --- | --- | --- |
| GCALL | 1 | 0.762** | 0.691** | 0.483** | 0.500** | -0.327* | -0.327* | 0.476** | -0.033 | 0.199 | 0.449** | 0.556** |
| GC1 | 0.762** | 1 | 0.389** | 0.052 | 0.266 | -0.184 | -0.193 | 0.272 | -0.084 | 0.075 | 0.351* | 0.419** |
| GC2 | 0.691** | 0.389** | 1 | -0.064 | 0.028 | 0.007 | -0.344** | 0.154 | -0.268 | 0 | 0.079 | 0.297* |
| GC3 | 0.483** | 0.052 | -0.064 | 1 | 0.702** | -0.474** | -0.096 | 0.513** | 0.307* | 0.325* | 0.449** | 0.364** |
| GC3s | 0.500** | 0.266 | 0.028 | 0.702** | 1 | -0.609** | -0.161 | 0.651** | 0.533** | 0.457** | 0.403** | 0.383** |
| A3 | -0.327* | -0.184 | 0.007 | -0.474** | -0.609** | 1 | -0.477** | -0.372** | -0.254 | -0.123 | -0.531** | -0.397** |
| T3 | -0.327* | -0.193 | -0.344** | -0.096 | -0.161 | -0.477** | 1 | -0.230 | 0.133 | -0.086 | 0.238 | -0.149 |
| C3 | 0.476** | 0.272 | 0.154 | 0.513** | 0.651** | -0.372** | -0.23 | 1 | -0.239 | 0.310* | 0.675** | 0.673** |
| G3 | -0.033 | -0.084 | -0.268 | 0.307* | 0.533** | -0.254 | 0.133 | -0.239 | 1 | 0.362** | -0.240 | -0.348* |
| ENC | 0.199 | 0.075 | 0 | 0.325* | 0.457** | -0.123 | -0.086 | 0.310* | 0.362** | 1 | 0.036 | -0.075 |
| CAI | 0.449** | 0.351* | 0.079 | 0.449** | 0.403** | -0.531** | 0.238 | 0.675** | -0.24 | 0.036 | 1 | 0.760** |
| CBI | 0.556** | 0.419** | 0.297* | 0.364** | 0.383** | -0.397** | -0.149 | 0.673** | -0.348* | -0.075 | 0.760** | 1 |

*A. sinomontanum*

|  | GC_ALL_ | GC_1_ | GC_2_ | GC_3_ | GC_3s_ | A_3_ | T_3_ | C_3_ | G_3_ | ENC | CAI | CBI |
| --- | --- | --- | --- | --- | --- | --- | --- | --- | --- | --- | --- | --- |
| GCALL | 1 | 0.781** | 0.756** | 0.521** | 0.478** | -0.332* | -0.266 | 0.440** | -0.012 | 0.248 | 0.351** | 0.463** |
| GC1 | 0.781** | 1 | 0.335* | 0.194 | 0.311* | -0.239 | -0.231 | 0.223 | 0.002 | 0.09 | 0.181 | 0.309* |
| GC2 | 0.756** | 0.335* | 1 | 0.138 | 0.071 | -0.047 | -0.243 | 0.190 | -0.237 | 0.074 | 0.132 | 0.289* |
| GC3 | 0.521** | 0.194 | 0.138 | 1 | 0.790** | -0.498** | -0.042 | 0.635** | 0.327* | 0.463** | 0.532** | 0.415** |
| GC3s | 0.478** | 0.311* | 0.071 | 0.790** | 1 | -0.575** | -0.188 | 0.645** | 0.533** | 0.519** | 0.358** | 0.393** |
| A3 | -0.332* | -0.239 | -0.047 | -0.498** | -0.575** | 1 | -0.495** | -0.322* | -0.245 | -0.160 | 0.272* | -0.149 |
| T3 | -0.266 | -0.231 | -0.243 | -0.042 | -0.188 | -0.495** | 1 | -0.273* | 0.127 | -0.129 | 0.272* | -0.149 |
| C3 | 0.440** | 0.223 | 0.19 | 0.635** | 0.645** | -0.322* | -0.273* | 1 | -0.246 | 0.340* | 0.620** | 0.687** |
| G3 | -0.012 | 0.002 | -0.237 | 0.327* | 0.533** | -0.245 | 0.127 | -0.246 | 1 | 0.404** | -0.238 | -0.351* |
| ENC | 0.248 | 0.09 | 0.074 | 0.463** | 0.519** | -0.16 | -0.129 | 0.340* | 0.404** | 1 | 0.014 | -0.039 |
| CAI | 0.351** | 0.181 | 0.132 | 0.532** | 0.358** | 0.272* | 0.272* | 0.620** | -0.238 | 0.014 | 1 | 0.754** |
| CBI | 0.463** | 0.309* | 0.289* | 0.415** | 0.393** | -0.149 | -0.149 | 0.687** | -0.351* | -0.039 | 0.754** | 1 |

*A. stapfianum*

|  | GC_ALL_ | GC_1_ | GC_2_ | GC_3_ | GC_3s_ | A_3_ | T_3_ | C_3_ | G_3_ | ENC | CAI | CBI |
| --- | --- | --- | --- | --- | --- | --- | --- | --- | --- | --- | --- | --- |
| GCALL | 1 | 0.805** | 0.782** | 0.527** | 0.507** | -0.343* | -0.342* | 0.420** | 0.026 | 0.253 | 0.364** | 0.428** |
| GC1 | 0.805** | 1 | 0.419** | 0.241 | 0.297* | -0.237 | -0.197 | 0.236 | -0.028 | 0.144 | 0.356** | 0.410** |
| GC2 | 0.782** | 0.419** | 1 | 0.132 | 0.111 | -0.032 | -0.387** | 0.162 | -0.180 | 0.051 | 0.067 | 0.259 |
| GC3 | 0.527** | 0.241 | 0.132 | 1 | 0.889** | -0.618** | -0.077 | 0.627** | 0.431** | 0.423** | 0.451** | 0.273* |
| GC3s | 0.507** | 0.297* | 0.111 | 0.889** | 1 | -0.615** | -0.167 | 0.644** | 0.543** | 0.471** | 0.339* | 0.258 |
| A3 | -0.343* | -0.237 | -0.032 | -0.618** | -0.615** | 1 | -0.474** | -0.340* | -0.295* | -0.148 | -0.522** | -0.303* |
| T3 | -0.342* | -0.197 | -0.387** | -0.077 | -0.167 | -0.474** | 1 | -0.273* | 0.157 | -0.116 | 0.280* | -0.139 |
| 8C3 | 0.420** | 0.236 | 0.162 | 0.627** | 0.644** | -0.340* | -0.273* | 1 | -0.240 | 0.375** | 0.607** | 0.605** |
| G3 | 0.026 | -0.028 | -0.18 | 0.431** | 0.543** | -0.295* | 0.157 | -0.24 | 1 | 0.295* | -0.254 | -0.426** |
| ENC | 0.253 | 0.144 | 0.051 | 0.423** | 0.471** | -0.148 | -0.116 | 0.375** | 0.295* | 1 | 0.071 | -0.007 |
| CAI | 0.364** | 0.356** | 0.067 | 0.451** | 0.339* | -0.522** | 0.280* | 0.607** | -0.254 | 0.071 | 1 | 0.724** |
| CBI | 0.428** | 0.410** | 0.259 | 0.273* | 0.258 | -0.303* | -0.139 | 0.605** | -0.426** | -0.007 | 0.724** | 1 |

*A. stylosum*

|  | GC_ALL_ | GC_1_ | GC_2_ | GC_3_ | GC_3s_ | A_3_ | T_3_ | C_3_ | G_3_ | ENC | CAI | CBI |
| --- | --- | --- | --- | --- | --- | --- | --- | --- | --- | --- | --- | --- |
| GCALL | 1 | 0.796** | 0.773** | 0.565** | 0.478** | -0.317* | -0.348* | 0.413** | 0.015 | 0.213 | 0.366** | 0.467** |
| GC1 | 0.796** | 1 | 0.389** | 0.248 | 0.270 | -0.219 | -0.196 | 0.213 | -0.029 | 0.108 | 0.370** | 0.402** |
| GC2 | 0.773** | 0.389** | 1 | 0.173 | 0.075 | -0.001 | -0.410** | 0.151 | -0.225 | 0.022 | 0.049 | 0.285* |
| GC3 | 0.565** | 0.248 | 0.173 | 1 | 0.863** | -0.586** | -0.105 | 0.646** | 0.416** | 0.421** | 0.437** | 0.329* |
| GC3s | 0.478** | 0.27 | 0.075 | 0.863** | 1 | -0.624** | -0.185 | 0.677** | 0.543** | 0.487** | 0.353** | 0.302* |
| A3 | -0.317* | -0.219 | -0.001 | -0.586** | -0.624** | 1 | -0.451** | -0.367** | -0.299* | -0.151 | -0.518** | -0.327* |
| T3 | -0.348* | -0.196 | -0.410** | -0.105 | -0.185 | -0.451** | 1 | -0.278* | 0.146 | -0.140 | 0.260 | -0.157 |
| 8C3 | 0.413** | 0.213 | 0.151 | 0.646** | 0.677** | -0.367** | -0.278* | 1 | -0.196 | 0.394** | 0.604** | 0.598** |
| G3 | 0.015 | -0.029 | -0.225 | 0.416** | 0.543** | -0.299* | 0.146 | -0.196 | 1 | 0.316* | -0.235 | -0.374** |
| ENC | 0.213 | 0.108 | 0.022 | 0.421** | 0.487** | -0.151 | -0.14 | 0.394** | 0.316* | 1 | 0.074 | -0.019 |
| CAI | 0.366** | 0.370** | 0.049 | 0.437** | 0.353** | -0.518** | 0.26 | 0.604** | -0.235 | 0.074 | 1 | 0.722** |
| CBI | 0.467** | 0.402** | 0.285* | 0.329* | 0.302* | -0.327* | -0.157 | 0.598** | -0.374** | -0.019 | 0.722** | 1 |

*A. tanguticum*

|  | GC_ALL_ | GC_1_ | GC_2_ | GC_3_ | GC_3s_ | A_3_ | T_3_ | C_3_ | G_3_ | ENC | CAI | CBI |
| --- | --- | --- | --- | --- | --- | --- | --- | --- | --- | --- | --- | --- |
| GCALL | 1 | 0.812** | 0.785** | 0.550** | 0.510** | -0.339* | -0.332* | -/448** | 0.014 | 0.280* | 0.376** | 0.497** |
| GC1 | 0.812** | 1 | 0.430** | 0.250 | 0.307* | -0.218 | -0.221 | 0.229 | -0.004 | 0.142 | 0.329* | 0.394** |
| GC2 | 0.785** | 0.430** | 1 | 0.162 | 0.105 | -0.039 | -0.393** | 0.201 | -0.238 | 0.081 | 0.090 | 0.354** |
| GC3 | 0.550** | 0.25 | 0.162 | 1 | 0.883** | -0.613** | -0.055 | 0.671** | 0.405** | 0.488** | 0.478** | 0.341* |
| GC3s | 0.510** | 0.307* | 0.105 | 0.883** | 1 | -0.634** | -0.132 | 0.663** | 0.543** | 0.544** | 0.351* | 0.309* |
| A3 | -0.339* | -0.218 | -0.039 | -0.613** | -0.634** | 1 | -0.477** | -0.364** | -0.307* | -0.165 | -0.510** | -0.334* |
| T3 | -0.332* | -0.221 | -0.393** | -0.055 | -0.132 | -0.477** | 1 | -0.236 | 0.182 | -0.162 | 0.297* | -0.144 |
| 8C3 | -/448** | 0.229 | 0.201 | 0.671** | 0.663** | -0.364** | -0.236 | 1 | -0.212 | 0.391** | 0.581** | 0.595** |
| G3 | 0.014 | -0.004 | -0.238 | 0.405** | 0.543** | -0.307* | 0.182 | -0.212 | 1 | 0.377** | -0.201 | -0.361** |
| ENC | 0.280* | 0.142 | 0.081 | 0.488** | 0.544** | -0.165 | -0.162 | 0.391** | 0.377** | 1 | 0.057 | -0.032 |
| CAI | 0.376** | 0.329* | 0.09 | 0.478** | 0.351* | -0.510** | 0.297* | 0.581** | -0.201 | 0.057 | 1 | 0.707** |
| CBI | 0.497** | 0.394** | 0.354** | 0.341* | 0.309* | -0.334* | -0.144 | 0.595** | -0.361** | -0.032 | 0.707** | 1 |

1. *vilmorinianum*

|  | GC_ALL_ | GC_1_ | GC_2_ | GC_3_ | GC_3s_ | A_3_ | T_3_ | C_3_ | G_3_ | ENC | CAI | CBI |
| --- | --- | --- | --- | --- | --- | --- | --- | --- | --- | --- | --- | --- |
| GCALL | 1 | 0.808** | 0.775** | 0.541** | 0.519** | -0.353** | -0.331* | 0.422** | 0.044 | 0.246 | 0.372** | 0.433** |
| GC1 | 0.808** | 1 | 0.418** | 0.241 | 0.295* | -0.236 | -0.201 | 0.238 | -0.032 | 0.145 | 0.356** | 0.412** |
| GC2 | 0.775** | 0.418** | 1 | 0.134 | 0.113 | -0.032 | -0.389** | 0.160 | -0.175 | 0.049 | 0.065 | 0.252 |
| GC3 | 0.541** | 0.241 | 0.134 | 1 | 0.889** | -0.618** | -0.079 | 0.624** | 0.431** | 0.420** | 0.449** | 0.267 |
| GC3s | 0.519** | 0.295* | 0.113 | 0.889** | 1 | -0.613** | -0.170 | 0.640** | 0.544** | 0.467** | 0.335* | 0.250 |
| A3 | -0.353** | -0.236 | -0.032 | -0.618** | -0.613** | 1 | -0.471** | -0.339* | -0.292* | -0.145 | -0.521** | -0.301* |
| T3 | -0.331* | -0.201 | -0.389** | -0.079 | -0.17 | -0.471** | 1 | -0.274* | 0.155 | -0.119 | 0.279* | -0.138 |
| 8C3 | 0.422** | 0.238 | 0.16 | 0.624** | 0.640** | -0.339* | -0.274* | 1 | -0.246 | 0.376** | 0.609** | 0.607** |
| G3 | 0.044 | -0.032 | -0.175 | 0.431** | 0.544** | -0.292* | 0.155 | -0.246 | 1 | 0.287* | -0.259 | -0.436** |
| ENC | 0.246 | 0.145 | 0.049 | 0.420** | 0.467** | -0.145 | -0.119 | 0.376** | 0.287* | 1 | 0.073 | -0.001 |
| CAI | 0.372** | 0.356** | 0.065 | 0.449** | 0.335* | -0.521** | 0.279* | 0.609** | -0.259 | 0.073 | 1 | 0.725** |
| CBI | 0.607** | -0.436** | -0.001 | 0.725** | 0.607** | -0.436** | -0.001 | 0.725** | 0.607** | -0.436** | -0.001 | 1 |

*A. weixiense*

|  | GC_ALL_ | GC_1_ | GC_2_ | GC_3_ | GC_3s_ | A_3_ | T_3_ | C_3_ | G_3_ | ENC | CAI | CBI |
| --- | --- | --- | --- | --- | --- | --- | --- | --- | --- | --- | --- | --- |
| GCALL | 1 | 0.809** | 0.775** | 0.540** | 0.520** | -0.352** | -0.325* | 0.426** | 0.043 | 0.247 | 0.379** | 0.445 |
| GC1 | 0.809** | 1 | 0.420** | 0.240 | 0.297* | -0.234 | -0.198 | 0.236 | -0.027 | 0.138 | 0.358** | 0.412** |
| GC2 | 0.775** | 0.420** | 1 | 0.132 | 0.111 | -0.032 | -0.386** | 0.165 | -0.183 | 0.053 | 0.071 | 0.266 |
| GC3 | 0.540** | 0.24 | 0.132 | 1 | 0.889** | -0.618** | -0.074 | 0.631** | 0.431** | 0.427** | 0.456** | 0.281* |
| GC3s | 0.520** | 0.297* | 0.111 | 0.889** | 1 | -0.615** | -0.164 | 0.49** | 0.543** | 0.476** | 0.345* | 0.269 |
| A3 | -0.352** | -0.234 | -0.032 | -0.618** | -0.615** | 1 | -0.478** | -0.341* | -0.298* | -0.152 | -0.525** | -0.306* |
| T3 | -0.325* | -0.198 | -0.386** | -0.074 | -0.164 | -0.478** | 1 | -0.274* | 0.163 | -0.112 | 0.278* | -0.143 |
| C3 | 0.426** | 0.236 | 0.165 | 0.631** | 0.49** | -0.341* | -0.274* | 1 | -0.234 | 0.375** | 0.605** | 0.604** |
| G3 | 0.043 | -0.027 | -0.183 | 0.431** | 0.543** | -0.298* | 0.163 | -0.234 | 1 | 0.306* | -0.245 | -0.425** |
| ENC | 0.247 | 0.138 | 0.053 | 0.427** | 0.476** | -0.152 | -0.112 | 0.375** | 0.306* | 1 | 0.072 | -0.014 |
| CAI | 0.379** | 0.358** | 0.071 | 0.456** | 0.345* | -0.525** | 0.278* | 0.605** | -0.245 | 0.072 | 1 | 0.725** |
| CBI | 0.445 | 0.412** | 0.266 | 0.281* | 0.269 | -0.306* | -0.143 | 0.604** | -0.425** | -0.014 | 0.725** | 1 |

|  | GC_ALL_ | GC_1_ | GC_2_ | GC_3_ | GC_3s_ | A_3_ | T_3_ | C_3_ | G_3_ | ENC | CAI | CBI |
| --- | --- | --- | --- | --- | --- | --- | --- | --- | --- | --- | --- | --- |
| GCALL | 1 | 0.821** | 0.766** | 0.529** | 0.517** | -0.372** | -0.26 | 0.437** | 0.027 | 0.257 | 0.428** | 0.452** |
| GC1 | 0.821** | 1 | 0.422** | 0.253 | 0.320* | -0.249 | -0.207 | 0.250 | -0.023 | 0.108 | 0.352** | 0.408** |
| GC2 | 0.766** | 0.422** | 1 | 0.104 | 0.110 | -0.064 | -0.293* | 0.182 | -0.194 | 0.092 | 0.138 | 0.268 |
| GC3 | 0.529** | 0.253 | 0.104 | 1 | 0.853** | -0.609** | -0.009 | 0.622** | 0.400** | 0.445** | 0.506** | 0.298* |
| GC3s | 0.517** | 0.320* | 0.110 | 0.853** | 1 | -0.618** | -0.152 | 0.658** | 0.523** | 0.479** | 0.361** | 0.279* |
| A3 | -0.372** | -0.249 | -0.064 | -0.609** | -0.618** | 1 | -0.479** | -0.346* | -0.289* | -0.159 | -0.536** | -0.308* |
| T3 | -0.26 | -0.207 | -0.293* | -0.009 | -0.152 | -0.479** | 1 | -0.283* | 0.192 | -0.085 | 0.280* | -0.149 |
| C3 | 0.437** | 0.250 | 0.182 | 0.622** | 0.658** | -0.346* | -0.283* | 1 | -0.245 | 0.394** | 0.603** | 0.598** |
| G3 | 0.027 | -0.023 | -0.194 | 0.400** | 0.523** | -0.289* | 0.192 | -0.245 | 1 | 0.300* | -0.237 | -0.410** |
| ENC | 0.257 | 0.108 | 0.092 | 0.445** | 0.479** | -0.159 | -0.085 | 0.394** | 0.300* | 1 | 0.086 | -0.008 |
| CAI | 0.428** | 0.352** | 0.138 | 0.506** | 0.361** | -0.536** | 0.280* | 0.603** | -0.237 | 0.086 | 1 | 0.720** |
| CBI | 0.452** | 0.408** | 0.268 | 0.298* | 0.279* | -0.308* | -0.149 | 0.598** | -0.410** | -0.008 | 0.720** | 1 |

*A. habaense*

|  | GC_ALL_ | GC_1_ | GC_2_ | GC_3_ | GC_3s_ | A_3_ | T_3_ | C_3_ | G_3_ | ENC | CAI | CBI |
| --- | --- | --- | --- | --- | --- | --- | --- | --- | --- | --- | --- | --- |
| GCALL | 1 | 0.808** | 0.775** | 0.522** | 0.489** | -0.326* | -0.299* | 0.454** | -0.013 | 0.243 | 0.411* | 0.474** |
| GC1 | 0.808** | 1 | 0.420** | 0.219 | 0.307* | -0.229 | -0.224 | 0.236 | -0.015 | 0.070 | 0.336* | 0.403** |
| GC2 | 0.775** | 0.420** | 1 | 0.113 | 0.075 | -0.010 | -0.336* | 0.201 | -0.262 | 0.077 | 0.123 | 0.296* |
| GC3 | 0.522** | 0.219 | 0.113 | 1 | 0.831** | -0.579** | -0.026 | 0.650** | 0.374** | 0.475** | 0.497** | 0.323* |
| GC3s | 0.489** | 0.307* | 0.075 | 0.831** | 1 | -0.630** | -0.145 | 0.665** | 0.543** | 0.512** | 0.335* | 0.300* |
| A3 | -0.326* | -0.229 | -0.010 | -0.579** | -0.630** | 1 | -0.474** | -0.353** | -0.317* | -0.179 | -0.503** | -0.321* |
| T3 | -0.299* | -0.224 | -0.336* | -0.026 | -0.145 | -0.474** | 1 | -0.257 | 0.183 | -0.089 | 0.288* | -0.145* |
| C3 | 0.454** | 0.236 | 0.201 | 0.650** | 0.665** | -0.353** | -0.257 | 1 | -0.210 | 0.390** | 0.571** | 0.588** |
| G3 | -0.013 | -0.015 | -0.262 | 0.374** | 0.543** | -0.317* | 0.183 | -0.210 | 1 | 0.365** | -0.220 | -0.370** |
| ENC | 0.243 | 0.070 | 0.077 | 0.475** | 0.512** | -0.179 | -0.089 | 0.390** | 0.365** | 1 | 0.032 | -0.060 |
| CAI | 0.411* | 0.336* | 0.123 | 0.497** | 0.335* | -0.503** | 0.288* | 0.571** | -0.220 | 0.032 | 1 | 0.725** |
| CBI | 0.474** | 0.403** | 0.296* | 0.323* | 0.300* | -0.321* | -0.145* | 0.588** | -0.370** | -0.060 | 0.725** | 1 |

*A. macrorhynchum*

|  | GC_ALL_ | GC_1_ | GC_2_ | GC_3_ | GC_3s_ | A_3_ | T_3_ | C_3_ | G_3_ | ENC | CAI | CBI |
| --- | --- | --- | --- | --- | --- | --- | --- | --- | --- | --- | --- | --- |
| GCALL | 1 | 0.807** | 0.705** | 0.524** | 0.486** | -0.331* | -0.227* | 0.449** | -0.018 | 0.248 | 0.418** | 0.483** |
| GC1 | 0.807** | 1 | 0.419** | 0.218 | 0.301* | -0.234 | -0.205 | 0.229 | -0.022 | 0.069 | 0.343* | 0.408** |
| GC2 | 0.705** | 0.419** | 1 | 0.118 | 0.078 | -0.026 | -0.315* | 0.201 | -0.264 | 0.088 | 0.130 | 0.305** |
| GC3 | 0.524** | 0.218 | 0.118 | 1 | 0.830** | -0.563** | -0.024 | 0.647** | 0.372** | 0.476** | 0.496** | 0.329* |
| GC3s | 0.486** | 0.301* | 0.078 | 0.830** | 1 | -0.608** | -0.146 | 0.667** | 0.540** | 0.507** | 0.336* | 0.307* |
| A3 | -0.331* | -0.234 | -0.026 | -0.563** | -0.608** | 1 | -0.504** | -0.333* | -0.309* | -0.178 | -0.521** | -0.365** |
| T3 | -0.227* | -0.205 | -0.315* | -0.024 | -0.146 | -0.504** | 1 | -0.253 | 0.172 | -0.083 | 0.312* | -0.086 |
| C3 | 0.449** | 0.229 | 0.201 | 0.647** | 0.667** | -0.333* | -0.253 | 1 | -0.212 | 0.387** | 0.561** | 0.569** |
| G3 | -0.018 | -0.022 | -0.264 | 0.372** | 0.540** | -0.309* | 0.172 | -0.212 | 1 | 0.353** | -0.215 | -0.342* |
| ENC | 0.248 | 0.069 | 0.088 | 0.476** | 0.507** | -0.178 | -0.083 | 0.387** | 0.353** | 1 | 0.044 | -0.039 |
| CAI | 0.418** | 0.343* | 0.130 | 0.496** | 0.336* | -0.521** | 0.312* | 0.561** | -0.215 | 0.044 | 1 | 0.735** |
| CBI | 0.483** | 0.408** | 0.305** | 0.329* | 0.307* | -0.365** | -0.086 | 0.569** | -0.342* | -0.039 | 0.735** | 1 |

*A. paniculigerum* var. *wulingense*

|  | GC_ALL_ | GC_1_ | GC_2_ | GC_3_ | GC_3s_ | A_3_ | T_3_ | C_3_ | G_3_ | ENC | CAI | CBI |
| --- | --- | --- | --- | --- | --- | --- | --- | --- | --- | --- | --- | --- |
| GCALL | 1 | 0.809** | 0.775** | 0.539** | 0.514** | -0.355** | -0.278* | 0.424** | 0.046 | 0.291* | 0.413** | 0.438** |
| GC1 | 0.809** | 1 | 0.419** | 0.239 | 0.310* | -0.245 | -0.205 | 0.238 | -0.019 | 0.136 | 0.351** | 0.408** |
| GC2 | 0.775** | 0.419** | 1 | 0.134 | 0.123 | -0.048 | -0.322* | 0.171 | -0.163 | 0.138 | 0.12 | 0.250 |
| GC3 | 0.539** | 0.239 | 0.134 | 1 | 0.840** | -0.590** | -0.023 | 0.614** | 0.402** | 0.432** | 0.492** | 0.292* |
| GC3s | 0.514** | 0.310* | 0.123 | 0.840** | 1 | -0.621** | -0.155 | 0.647** | 0.541** | 0.468** | 0.349* | 0.275* |
| A3 | -0.355** | -0.245 | -0.048 | -0.590** | -0.621** | 1 | -0.478** | -0.344* | -0.301* | -0.156 | -0.524** | -0.306* |
| T3 | -0.278* | -0.205 | -0.322* | -0.023 | -0.155 | -0.478** | 1 | -0.268 | 0.171 | -0.096 | 0.277* | -0.147 |
| C3 | 0.424** | 0.238 | 0.171 | 0.614** | 0.647** | -0.344* | -0.268 | 1 | -0.238 | 0.368** | 0.608** | 0.608** |
| G3 | 0.046 | -0.019 | -0.163 | 0.402** | 0.541** | -0.301* | 0.171 | -0.238 | 1 | 0.303* | -0.242 | -0.411** |
| ENC | 0.291* | 0.136 | 0.138 | 0.432** | 0.468** | -0.156 | -0.096 | 0.368** | 0.303* | 1 | 0.077 | -0.011 |
| CAI | 0.413** | 0.351** | 0.12 | 0.492** | 0.349* | -0.524** | 0.277* | 0.608** | -0.242 | 0.077 | 1 | 0.724** |
| CBI | 0.438** | 0.408** | 0.250 | 0.292* | 0.275* | -0.306* | -0.147 | 0.608** | -0.411** | -0.011 | 0.724** | 1 |

*A. tanssectum*

|  | GC_ALL_ | GC_1_ | GC_2_ | GC_3_ | GC_3s_ | A_3_ | T_3_ | C_3_ | G_3_ | ENC | CAI | CBI |
| --- | --- | --- | --- | --- | --- | --- | --- | --- | --- | --- | --- | --- |
| GCALL | 1 | 0.809** | 0.774** | 0.458** | 0.483** | 0.322* | -0.292* | 0.449** | -0.016 | 0.252 | 0.408** | 0.470** |
| GC1 | 0.809** | 1 | 0.422** | 0.206 | 0.295* | -0.221 | -0.22 | 0.233 | -0.029 | 0.087 | 0.337* | 0.399** |
| GC2 | 0.774** | 0.422** | 1 | 0.058 | 0.072 | -0.010 | -0.331* | 0.196 | -0.257 | 0.071 | 0.121 | 0.298* |
| GC3 | 0.458** | 0.206 | 0.058 | 1 | 0.807** | -0.523** | -0.095 | 0.667** | 0.308* | 0.432** | 0.452** | 0.320* |
| GC3s | 0.483** | 0.295* | 0.072 | 0.807** | 1 | -0.625** | -0.135 | 0.659** | 0.544** | 0.510** | 0.330* | 0.294* |
| A3 | 0.322* | -0.221 | -0.010 | -0.523** | -0.625** | 1 | -0.488** | -0.353** | -0.306* | -0.166 | -0.502** | -0.324* |
| T3 | -0.292* | -0.22 | -0.331* | -0.095 | -0.135 | -0.488** | 1 | -0.242 | 0.172 | -0.096 | 0.294* | -0.133 |
| C3 | 0.449** | 0.233 | 0.196 | 0.667** | 0.659** | -0.353** | -0.242 | 1 | -0.218 | 0.384** | 0.577** | 0.586** |
| G3 | -0.016 | -0.029 | -0.257 | 0.308* | 0.544** | -0.306* | 0.172 | -0.218 | 1 | 0.361** | -0.234 | -0.372** |
| ENC | 0.252 | 0.087 | 0.071 | 0.432** | 0.510** | -0.166 | -0.096 | 0.384** | 0.361** | 1 | 0.029 | -0.067 |
| CAI | 0.408** | 0.337* | 0.121 | 0.452** | 0.330* | -0.502** | 0.294* | 0.577** | -0.234 | 0.029 | 1 | 0.724** |
| CBI | 0.470** | 0.399** | 0.298* | 0.320* | 0.294* | -0.324* | -0.133 | 0.586** | -0.372** | -0.067 | 0.724** | 1 |

*A. tschangbaischanense*

|  | GC_ALL_ | GC_1_ | GC_2_ | GC_3_ | GC_3s_ | A_3_ | T_3_ | C_3_ | G_3_ | ENC | CAI | CBI |
| --- | --- | --- | --- | --- | --- | --- | --- | --- | --- | --- | --- | --- |
| GCALL | 1 | 0.781** | 0.718** | 0.429** | 0.485** | -0.324* | -0.261 | 0.449** | -0.003 | 0.290* | 0.437** | 0.525** |
| GC1 | 0.781** | 1 | 0.388** | 0.064 | 0.293* | -0.234 | -0.148 | 0.344* | -0.103 | 0.148 | 0.391** | 0.442** |
| GC2 | 0.718** | 0.388** | 1 | -0.078 | 0.034 | -0.065 | -0.207 | 0.203 | -0.279* | 0.203 | 0.124 | 0.274* |
| GC3 | 0.429** | 0.064 | -0.078 | 1 | 0.705** | -0.369** | -0.158 | 0.347* | 0.467** | 0.229 | 0.359** | 0.314* |
| GC3s | 0.485** | 0.293* | 0.034 | 0.705** | 1 | -0.575** | -0.182 | 0.644** | 0.558** | 0.539** | 0.347* | 0.364** |
| A3 | -0.324* | -0.234 | -0.065 | -0.369** | -0.575** | 1 | -0.507** | -0.320* | -0.272* | -0.17 | -0.497** | -0.353** |
| T3 | -0.261 | -0.148 | -0.207 | -0.158 | -0.182 | -0.507** | 1 | -0.251 | 0.111 | -0.162 | 0.279* | -0.13 |
| C3 | 0.449** | 0.344* | 0.203 | 0.347* | 0.644** | -0.320* | -0.251 | 1 | -0.22 | 0.374** | 0.615** | 0.666**** |
| G3 | -0.003 | -0.103 | -0.279* | 0.467** | 0.558** | -0.272* | 0.111 | -0.22 | 1 | 0.383** | -0.231 | -0.345* |
| ENC | 0.290* | 0.148 | 0.203 | 0.229 | 0.539** | -0.17 | -0.162 | 0.374** | 0.383** | 1 | 0.014 | -0.222 |
| CAI | 0.437** | 0.391** | 0.124 | 0.359** | 0.347* | -0.497** | 0.279* | 0.615** | -0.231 | 0.014 | 1 | 0.751** |
| CBI | 0.525** | 0.442** | 0.274* | 0.314* | 0.364** | -0.353** | -0.13 | 0.666**** | -0.345* | -0.222 | 0.751** | 1 |

*A. umbrosum*

|  | GC_ALL_ | GC_1_ | GC_2_ | GC_3_ | GC_3s_ | A_3_ | T_3_ | C_3_ | G_3_ | ENC | CAI | CBI |
| --- | --- | --- | --- | --- | --- | --- | --- | --- | --- | --- | --- | --- |
| GCALL | 1 | 0.807** | 0.775** | 0.523** | 0.488** | -0.326* | -0.296* | 0.452** | -0.01 | 0.255 | 0.410** | 0.480** |
| GC1 | 0.807** | 1 | 0.421** | 0.216 | 0.299* | -0.224 | -0.223 | 0.232 | -0.021 | 0.079 | 0.338 | 0.408** |
| GC2 | 0.775** | 0.421** | 1 | 0.114 | 0.077 | -0.013 | -0.334* | 0.201 | -0.257 | 0.081 | 0.2121 | 0.299* |
| GC3 | 0.523** | 0.216 | 0.114 | 1 | 0.833** | -0.579** | -0.024 | 0.648** | 0.381 | 0.489** | 0.494** | 0.329* |
| GC3s | 0.488** | 0.299* | 0.077 | 0.833** | 1 | -0.630** | -0.141 | 0.665** | 0.547 | 0.521** | 0.334* | 0.306* |
| A3 | -0.326* | -0.224 | -0.013 | -0.579** | -0.630** | 1 | -0.478** | -0.352** | -0.32 | -0.186 | ’-0.502** | -0.330* |
| T3 | -0.296* | -0.223 | -0.334* | -0.024 | -0.141 | -0.478** | 1 | -0.257 | 0.183 | -0.079 | 0.291* | -0.141 |
| C3 | 0.452** | 0.232 | 0.201 | 0.648** | 0.665** | -0.352** | -0.257 | 1 | -0.206 | 0.385** | 0.570** | 0.591** |
| G3 | -0.01 | -0.021 | -0.257 | 0.381 | 0.547 | -0.32 | 0.183 | -0.206 | 1 | 0.381** | -0.219 | -0.364** |
| ENC | 0.255 | 0.079 | 0.081 | 0.489** | 0.521** | -0.186 | -0.079 | 0.385** | 0.381** | 1 | 0.036 | -0.067 |
| CAI | 0.410** | 0.338 | 0.2121 | 0.494** | 0.334* | ’-0.502** | 0.291* | 0.570** | -0.219 | 0.036 | 1 | 0.728** |
| CBI | 0.480** | 0.408** | 0.299* | 0.329* | 0.306* | -0.330* | -0.141 | 0.591** | -0.364** | -0.067 | 0.728** | 1 |

*A. volubile*
